# ﻿Integrative taxonomy reveals two new giant pill-millipedes of the genus *Zephronia* Gray, 1832 from eastern Thailand (Diplopoda, Sphaerotheriida, Zephroniidae)

**DOI:** 10.3897/zookeys.1212.126536

**Published:** 2024-09-13

**Authors:** Ruttapon Srisonchai, Natdanai Likhitrakarn, Chirasak Sutcharit, Thomas Wesener

**Affiliations:** 1 Department of Biology, Faculty of Science, Khon Kaen University, Khon Kaen 40002, Thailand Khon Kaen University Khon Kaen Thailand; 2 Program of Agriculture, Faculty of Agricultural Production, Maejo University, Chiang Mai 50290, Thailand Maejo University Chiang Mai Thailand; 3 Animal Systematics Research Unit, Department of Biology, Faculty of Science, Chulalongkorn University, Phayathai Road, Patumwan, Bangkok 10330, Thailand Chulalongkorn University Bangkok Thailand; 4 Zoological Research Museum Alexander Koenig, Leibniz Institute for the Study of Biodiversity Change (LIB), Adenauerallee 160, D-53113 Bonn, Germany Zoological Research Museum Alexander Koenig, Leibniz Institute for the Study of Biodiversity Change (LIB) Bonn Germany

**Keywords:** Biodiversity, limestone, Myriapoda, Southeast Asia, taxonomy

## Abstract

A large amount of material of the millipede genus *Zephronia* Gray, 1832 was collected during 2014–2023 from many parts of eastern Thailand. An integrative study of morphological characters and genetic data (COI gene) revealed two new species: *Z.chantaburiensis* Srisonchai & Wesener, **sp. nov.** and *Z.macula* Srisonchai & Wesener, **sp. nov.** The two new species clearly differ from other congeners by their unique characteristics, especially in their colour pattern and telopod shape. The interspecific genetic distances of the 658 bp COI gene barcoding fragment between these new species and all other species of giant pill-millipede from Thailand, Laos and Cambodia are 12.01–23.49% for *Z.chantaburiensis***sp. nov.** and 17.93–25.13% for *Z.macula***sp. nov.** While relationships among species remain preliminary, the phylogenetic tree shows that species of *Zephronia* are interspersed with species of *Sphaerobelum* Verhoeff, 1924 and *Prionobelum* Verhoeff, 1924. Phylogenetic analyses place both new species in a clade termed *Zephronia* s.s., which receives support also from morphological data, showing a unique position of the organ of Tömösváry. *Z.macula***sp. nov.** appears to occur over a broad distribution whereas *Z.chantaburiensis***sp. nov.** was found only at the type locality. Given that all known records are in the eastern part of Thailand, we thus regard both species as endemic. Morphological illustrations based on SEM micrographs and a distribution map are also provided.

## ﻿Introduction

Intensive research on the millipedes (class Diplopoda) in Thailand began in 2007 after the discovery of a beautifully ornamented and endemic creature, the shocking pink dragon millipede *Desmoxytespurpurosea* Enghoff, Sutcharit & Panha, 2007 which has been endorsed by the public and has inspired further studies on several millipede groups (Enghoff et al. 2007; Likhitrakarn et al. 2023). A large number of newly discovered species and unidentified specimens has become available since then, and many of these were recently described by conventional taxonomic methods and included in phylogenetic analyses (i.e., Likhitrakarn et al. 2011; Srisonchai et al. 2016; Pimvichai et al. 2020). Thailand presents the highest number of described diplopod species for SE Asia today, accounting for more than 264 of 500 known species in mainland Southeast Asia (Nguyen and Sierwald 2013; Likhitrakarn et al. 2023), with striking genera and species discovered in many underexplored locations of Thailand and even in anthropogenically modified habitats.

Currently, three genera of giant pill-millipedes (order Sphaerotheriida) are known from Thailand: *Sphaerobelum* Verhoeff, 1924 with four species (see Srisonchai et al. 2023), *Prionobelum* Verhoeff, 1924 with two species (see Donworth and Wesener 2024), and *Zephronia* Gray, 1832 with nine species (see Bhansali and Wesener 2022). Generally, *Zephronia* is one of the most speciose groups among the diplopods and contains more than 50 described species (Wesener 2016). Recent research has continued to underscore the remarkably high diversity within the genus *Zephronia*, which has led to the discovery of an increasing number of species (Golovatch et al. 2012; Semenyuk et al. 2018; Wesener 2019; Likhitrakarn et al. 2021; Srisonchai et al. 2021; Rosenmejer et al. 2021; Bhansali and Wesener 2022; Zhao et al. 2022). A total of nine species of *Zephronia* have been recorded so far from Thailand (see Likhitrakarn et al. 2023), while only one species is known from Cambodia (*Z.dawydoffi* Attems, 1953) and one from Laos (*Z.laotica* Wesener, 2019). However, as with other closely related genera within the family, taxonomic studies on the genus *Zephronia* have posed intriguing challenges due to the insufficient genetic information and unknown evolutionary relationships (Wesener 2019).

The genetic approach based on DNA barcoding has been widely used for species delimitation in millipedes in recent years, but it is considered more reliable when used in combination with morphological evidence. Previous phylogenetic studies have notably provided insights into species discrimination for *Zephronia* (Wesener 2019; Rosenmejer et al. 2021; Bhansali and Wesener 2022; Zhao et al. 2022).

Considering the newly collected material, two morphologically distinct groups of specimens were observed during fieldwork. Given their visible morphological differences from the other known Thai giant pill-millipede species, the suspicion arose that they may represent undescribed species. In this paper, we integratively describe two new species from the eastern part of Thailand based on morphological traits using scanning electron microscopy and phylogenetic analyses based on mitochondrial COI sequences, in order to confirm their status and compare them with the known congeneric species from Thailand, Laos and Cambodia.

## ﻿Material and methods

### ﻿Specimen collections

The material was obtained from the collections of the Chulalongkorn University Museum of Zoology (**CUMZ**). New sampling was also conducted throughout the eastern part of Thailand and the surrounding areas in 2022–2023. All individuals were collected by hand and some of them were photographed alive in the field using a Canon 90D digital camera equipped with a Canon EF 100 mm f/2.8 Macro USM lens. Animal euthanasia was applied during the management of specimens based on AVMA guidelines (American Veterinary Medical Association 2020). The research protocol was approved by the Institutional Animal Care and Use Committee, Khon Kaen University (No. IACUC-KKU-136/64). The studied specimens were preserved in 70% ethanol for morphological examination and in 95% ethanol for molecular analyses. The data of GPS and habitats were also recorded.

### ﻿Maps

The map present herein was generated based on the background photo from the Elastic Terrain Map (Willett et al. 2015), and edited in Adobe Photoshop CS6.

### ﻿Morphological identifications

The format of the morphological descriptions follows Golovatch et al. (2012), Wongthamwanich et al. (2012), Wesener (2019), and Semenyuk et al. (2018). More than 600 specimens were examined under the stereo microscope. All morphological characters were thoroughly compared with the previous descriptions and compared with some of the available type specimens or topotypes.

The holotypes, as well as the paratypes, are deposited in the Chulalongkorn University Museum of Zoology, Thailand (**CUMZ**), the Natural History Museum of Denmark, Denmark (**NHMD**), the Naturhistorisches Museum Wien, Austria (**NHMW**) and the Zoological Research Museum Koenig, Germany (**ZFMK**).

### ﻿Abbreviations used in this study

List of abbreviations used in the description and figures:

**3it** 3-combed inner tooth of mandible,

**bu** bursa of vulva,

**co** condylus of mandible,

**cp** cuticular impression of endotergum;

**Cp** central pad of gnathochilarium,

**cr-t** crenulated teeth on telopods,

**ct** central tooth,

**cx** coxa,

**et** external tooth of epipharynx,

**Et** external tooth of mandibular gnathal lobe,

**fe** femur,

**ia** inner area of endotergum;

**Ia** inner area of mandible,

**il** incisura lateralis of the head,

**imf** immovable finger of telopod;

**ip** inner palpi of gnathochilarium,

**ll** lamellae lingulales of gnathochilarium,

**ma** middle area of endotergum;

**me** mentum of gnathochilarium,

**ml** membranous lobe,

**mp** molar plate of mandible,

**op** operculum of vulva,

**pl** pectinate lamellae of mandible,

**pm** posterior margin of endotergum;

**po** postfemur,

**pre** prefemur,

**rsp** row of spines,

**sc** sensory cone,

**scl-s** sclerotized spots,

**st** stipites of gnathochilarium,

**st-pl** stigmatic plate,

**ta** tarsus,

**ti** tibia.

Specimen repositories and others

**CUMZ**Chulalongkorn University Museum of Zoology, Thailand;

**KKUMZ**Khon Kaen University Museum of Zoology, Thailand;

**NHMD** Natural History Museum of Denmark, Denmark;

**NHMW**Naturhistorisches Museum Wien, Vienna, Austria;

**ZFMK**Zoological Research Museum Koenig, Bonn, Germany.

### ﻿Scanning electron microscope

Body parts of specimens for scanning electron microscopy (SEM) were carefully dissected under a stereomicroscope, placed in a dry cabinet for 24 hours, mounted on aluminium stubs, and then coated with gold. Objects were examined under a high vacuum in JEOL, JSM-5410 LV at the faculty of Science, Khon Kaen University. All figures were assembled and adjusted in Adobe Photoshop CS6.

### ﻿DNA extraction and PCR amplifications

DNA of 11 specimens (9 specimens of the two new species and 2 specimens of *Z.siamensis*) were extracted from the legs using the NucleoSpin Tissue Kit. We analysed the mitochondrial cytochrome c oxidase subunit I (COI) as a DNA barcoding gene. The polymerase chain reactions were carried out using LCO-1490 as forward and HCO-2198 as reverse primers — LCO-1490 (5'-GGT CAA CAA ATC ATA AAG ATA TTG G-3') and HCO-2198 (5'-TAA ACT TCA GGG TGA CCA AAA AAT CA-3') (Folmer et al. 1994). A total volume of 30.0 µL including 1.0 µL of DNA template — 43 °C for 2 minutes as annealing step and 72 °C for 2 minutes as an extension step. The target gene was verified by 1% agarose gel electrophoresis and eventually observed under UV trans-illumination. PCR products of COI were sequenced externally at Bioneers Co. (Korea).

### ﻿DNA alignment and phylogenetic reconstruction

We analysed a total of 84 sequences comprising 11 sequences generated from this study and 73 sequences obtained from GenBank, including all known giant pill-millipede species of *Zephronia* from Thailand, Cambodia, and Laos. *Sphaerobelum* spp., *Prionobelum* spp., *Cryxusovalis* (Linnaeus, 1758), *Epicyliosoma* sp., *Arthrosphaerabrandti* (Humbert, 1865), *Sphaeromimussplendidus* Wesener & Sierwald, 2005 and *Glomerismarginata* (Villers, 1789) were used as outgroups. All new sequences were submitted to GenBank with accession numbers provided in Table 1.

**Table 1.** Lists of *Zephronia*, *Sphaerobelum*, *Prionobelum* and *Cryxus* species analysed in this study and their COI accession numbers.

	Species	COI accession number	Voucher code	Locality	Reference
**Ingroups**					
1	*Z.chantaburiensis* sp. nov.	PP754582	CUMZ (M47)	Thailand, Chantaburi Province, Tha Mai District, Wat Khao Sukim	This study
2	*Z.chantaburiensis* sp. nov.	PP754583	CUMZ (M48)	Thailand, Chantaburi Province, Tha Mai District, Wat Khao Sukim	This study
3	*Z.chrysomallos* Bhansali & Wesener, 2022	OM509649	ZFMK MYR8826	Thailand, Kanchanaburi Province, Sai Yok District, Sai Yok Noi Waterfall	Bhansali and Wesener 2022
4	*Z.dawydoffi* Attems, 1953	MK330971	ZFMK Myr4504	N/A	Wesener 2019
5	*Z.erawani* Bhansali & Wesener, 2022	OM509650	NHMD K56x9	Thailand, Kanchanaburi Province, Si Sawat District, 50 km W of Kanchanaburi, Erawan Waterfall	Bhansali and Wesener 2022
6	*Z.golovatchi* Srisonchai, Sutcharit & Likhitrakarn, 2021	OM509646	ZFMK MYR6262	Thailand, Nakhon Ratchasima Province, Pak Chong District	Bhansali and Wesener 2022
7	*Z.golovatchi* Srisonchai, Sutcharit & Likhitrakarn, 2021	OM509647	ZFMK K53	Thailand, Nakhon Nayok Province, Khao Yai National Park	Bhansali and Wesener 2022
8	*Z.hui* Liu & Wesener, 2022	OP339790	SCAU YGM03	China, Guizhou, Tongren City, Jiangkou County, Taiping Town, Yamugou Scenic Area	Zhao et al. 2022
9	*Z.hui* Liu & Wesener, 2022	OP339791	SCAU YGM02	China, Guizhou, Tongren City, Jiangkou County, Taiping Town, Yamugou Scenic Area	Zhao et al. 2022
10	*Z.lannaensis* Likhitrakarn & Golovatch, 2021	OM509629	ZFMK MYR3498	Thailand, Chiangmai Province, Mueang District, Doi Suthep	Bhansali and Wesener 2022
11	*Z.lannaensis* Likhitrakarn & Golovatch, 2021	OM509630	ZFMK MYR3501	Thailand, Chiangmai Province, Mae Rim District, Traidhos School Campus	Bhansali and Wesener 2022
12	*Z.lannaensis* Likhitrakarn & Golovatch, 2021	OM509631	ZFMK MYR4911	Thailand, Chiangmai Province, Mae Rim District, Mae Sa Valley	Bhansali and Wesener 2022
13	*Z.lannaensis* Likhitrakarn & Golovatch, 2021	OM509632	NHMD K57B	Thailand, Chiangmai Province, Mueang District, Doi Suthep, Me Sa Waterfall	Bhansali and Wesener 2022
14	*Z.lannaensis* Likhitrakarn & Golovatch, 2021	OM509633	MHNG 3B	Thailand, Chiangmai Province, Mueang District, Doi Suthep	Bhansali and Wesener 2022
15	*Z.laotica* Wesener, 2019	MK330977	ZFMK Myr3502	Laos, Champasak Province, east of Mekong, Garden of Erawan Riverside Hotel	Wesener 2019
16	*Z.macula* sp. nov.	PP754589	CUMZ (M54)	Thailand, Sra Kaeo Province, Mueang Sra Kaeo District, Wat Tham Khao Maka	This study
17	*Z.macula* sp. nov.	PP754590	CUMZ (M55)	Thailand, Sra Kaeo Province, Mueang Sra Kaeo District, Wat Tham Khao Maka	This study
18	*Z.macula* sp. nov.	PP754584	CUMZ (M417)	Thailand, Chantaburi Province, Khlung District, Thaeo Khlong Khlung Monastery	This study
19	*Z.macula* sp. nov.	PP754585	CUMZ (M418)	Thailand, Chantaburi Province, Khlung District, Thaeo Khlong Khlung Monastery	This study
20	*Z.macula* sp. nov.	PP754586	CUMZ (M421)	Thailand, Chantaburi Province, Tha Mai District, Wat Khao Sukim	This study
21	*Z.macula* sp. nov.	PP754587	CUMZ (M422)	Thailand, Chantaburi Province, Tha Mai District, Wat Khao Sukim	This study
22	*Z.macula* sp. nov.	PP754588	CUMZ (M424)	Thailand, Chantaburi Province, Tha Mai District, Wat Khao Sukim	This study
23	*Z.medongensis* Zhao & Liu, 2022	OP339793	SCAU XZ01	China, Xizang Autonomous Region, Medog County	Zhao et al. 2022
24	*Z.ovalis* Gray, 1832	JX486068	ZFMK Myr 0832	Vietnam, Dong Nai Province, Cat Tien National Park	Golovatch et al. 2012
25	*Z.panhai* Srisonchai, Sutcharit & Likhitrakarn, 2021	OM509643	ZFMK MYR 8118	Thailand, Ratchaburi Province, Ratchaburi-Photharam Districts	Bhansali and Wesener 2022
26	*Z.panhai* Srisonchai, Sutcharit & Likhitrakarn, 2021	OM509644	MHNG 3A	Thailand, Ratchaburi Province, Chom Bueang District, Tham Kao Bin Forest Park	Bhansali and Wesener 2022
27	*Z.panhai* Srisonchai, Sutcharit & Likhitrakarn, 2021	OM509645	ZFMK MYR 8116	Thailand, Ratchaburi Province, Ratchaburi-Photharam Districts	Bhansali and Wesener 2022
28	*Z.phrain* Likhitrakarn & Golovatch, 2021	OM509634	ZFMK MYR 3499	Thailand, Chiang Mai Province, Chiang Dao District, Padeng Lodge	Bhansali and Wesener 2022
29	*Z.phrain* Likhitrakarn & Golovatch, 2021	OM509635	MYR3500	Thailand, Chiangmai Province, Mueang District, Doi Suthep	Bhansali and Wesener 2022
30	*Z.phrain* Likhitrakarn & Golovatch, 2021	OM509636	SMF	Thailand, Chiang Mai Province, Chiang Dao District, Tham Houay Luk	Bhansali and Wesener 2022
31	*Z.phrain* Likhitrakarn & Golovatch, 2021	OM509637	SMF	Thailand, Chiang Mai Province, Chiang Dao District, Doi Chiang Dao, Ma Lee’s Resort	Bhansali and Wesener 2022
32	*Z.phrain* Likhitrakarn & Golovatch, 2021	OM509638	SMF	Thailand, Chiang Mai Province, Chai Prakan District, Tham Ngam	Bhansali and Wesener 2022
33	*Z.phrain* Likhitrakarn & Golovatch, 2021	OM509639	ZFMK MYR 4907	Thailand, Chiang Mai Province, Chiang Dao	Bhansali and Wesener 2022
34	*Z.phrain* Likhitrakarn & Golovatch, 2021	OM509640	MHNG 5G	Thailand, Lamphun Province, Mae Tha District, Doi Khuntan National Park	Bhansali and Wesener 2022
35	*Z.phrain* Likhitrakarn & Golovatch, 2021	OM509641	MHNG 5I	Thailand, Chiang Mai Province, Chiang Dao District, Doi Chiang Dao	Bhansali and Wesener 2022
36	*Z.phrain* Likhitrakarn & Golovatch, 2021	OM509642	NHMD K35	Thailand, Chiang Mai Province, Ban Musue	Bhansali and Wesener 2022
37	*Z.siamensis* Hirst, 1907	JX486067.2	FMNH-INS-72669	Thailand, Chonburi Province, Sichang District, Koh Sichang	Golovatch et al.2012
38	*Z.siamensis* Hirst, 1907	OR530089	CUMZ	Thailand, Chonburi Province, Sichang District, Koh Sichang	Srisonchai et al. 2023
39	*Z.siamensis* Hirst, 1907	PP754592	CUMZ (M455)	Thailand, Chachoengsao Province, Phanom Sarakham District, Wat Khao Hin Sorn	This study
40	*Z.siamensis* Hirst, 1907	PP754591	CUMZ (D397)	Thailand, Srakaeo Province, Khao Chakan District, Wat Tham Khao Chan	This study
41	*Zephronia* sp. (K45 Aow Noi Temple)	MW898741	NHMD K55	Thailand, Prachuap Kiri Khan Province, Mueang District, Aow Noi Temple	Rosenmejer et al. 2021
42	*Zephronia* sp. (Aow Noi Temple)	MW898742	ZFMK MYR 8787	Thailand, Prachuap Kiri Khan Province, Mueang District, Aow Noi Temple	Rosenmejer et al. 2021
43	*Z.viridisoma* Rosenmejer & Wesener, 2021	MW898739	NHMD 621695	Thailand, Nakhon Si Thammarat Province, Sichon District, Khao Lark Waterfall	Rosenmejer et al. 2021
44	*Z.viridisoma* Rosenmejer & Wesener, 2021	MW898740	ZFMK MYR 8822	Thailand, Nakhon Si Thammarat Province, Sichon District, Khao Lark Waterfall	Rosenmejer et al. 2021
45	*Z.zhouae* Zhao & Liu, 2022	OP339794	SCAU YN02	China, Yunnan Province, Diqing Tibetan Autonomous Prefecture, Weixi County, Laowo Village	Rosenmejer et al. 2021
**Outgroups**					
46	*S.aesculus* Rosenmejer & Wesener, 2021	MW898737	NHMD 621693	Thailand, Phuket Province, Kathu District, Forest	Rosenmejer et al.2021
47	S.cf.aesculus Rosenmejer & Wesener, 2021	MW898738	NHMD 621694	Thailand, Nakhon Si Thammarat Province, Khao Luang NP	Rosenmejer et al. 2021
48	*S.benquii* Liu & Wesener, 2022	OP339792	SCAU MMY01	China, Guizhou, Tongren City, Jiangkou County, Guanhe Town, Guanhe Village, Maomaoyan	Zhao et al. 2022
49	*S.bolavensis* Wesener, 2019	MK330982	MHNG LT-10/24	Laos, Champasak Province, Bolaven Plateau, 3 km S of Ban Nong Luang, Tad Kameud	Wesener 2019
50	*S.denticulatum* Wesener, 2019	MK330984	MHNG LT-10/12	Laos, Oudomxai Province, ca 3 km E of Tad Lak 11, SE of Oudomxai city	Wesener 2019
51	*S.huzhengkuni* Zhao, Yu & Liu, 2020	MT657327	SCAU SP02	China, Guizhou Province, Tongren City, Fanjingshan National Nature Reserve	Zhao et al. 2020
52	*S.huzhengkuni* Zhao, Yu & Liu, 2020	MT657328	SCAU SP03	China, Guizhou Province, Tongren City, Fanjingshan National Nature Reserve	Zhao et al. 2020
53	*S.lachneeis* Wesener, 2019	MK330983	MHNG LT-10/12	Laos, Oudomxai Province, ca 3 km E of Tad Lak 11, SE of Oudomxai city	Wesener 2019
54	*S.laoticum* Wesener, 2019	MK330975	SMF	Laos, Vientiane Province, Vang Vieng	Wesener 2019
55	*S.meridionalis* Bhansali & Wesener, 2022	OM509648	MHNG 4B-2	Thailand, Yala Province, Bannang Sata District, Bang Lang National Park, near Than To Waterfall	Bhansali and Wesener 2022
56	*S.nigrum* Wesener, 2019	MK330976	SMF	Laos, Champasak Province, Muang Bachieng, Ban Lak 35, Tad Etu	Wesener 2019
57	*S.peterjaegeri* Wesener, 2019	MK330972	SMF SD553	Laos, Luang Prabang Province, SE Luang Prabang, Nam Khan, Ban Pak Bak, Houay Kho	Wesener 2019
58	*S.phouloei* Wesener, 2019	MK330974	ZMUC00040257	Laos, Houaphan Province, Phou Loei	Wesener 2019
59	*S.schwendingeri* Wesener, 2019	MK330978	MHNG LT-10/03	Laos, Vientiane Province, trail to Tham Pou Kham, W. of Vang Vieng	Wesener 2019
60	*S.schwendingeri* Wesener, 2019	MK330981	SMF	Laos, Vientiane Province, Vang Vieng, Tham Pou Kham	Wesener 2019
61	*Sphaerobelum* sp. L07	MK330979	ZMUC00040261	Laos, Khammouane Province, Ban Khounkham [Khun Kham] (Nahin)	Wesener 2019
62	*Sphaerobelum* sp. L10	MK330980	SMF	Laos, Vientiane Province, Vang Vieng, W. of Nam Song, Tham Nam Or Khem	Wesener 2019
63	*S.spinatum* Wesener, 2019	MK330973	ZMUC00040258	Laos, Vientiane Province, Phou Khao Khouay	Wesener 2019
64	*S.truncatum* Wongthamwanich, 2012	JN885184	FMNH-INS 0000 072 674	Thailand, Nan Province, Song Khwae District, Na Rai Luang Subdistrict, Pang Hi Village	Wongthamwanich et al. 2012
65	*S.turcosa* Srisonchai & Pimvichai, 2023	OR530087	CUMZ-Zeph0012	Thailand, Loei Province, Mueang Loei District, Phu Pha Lom Forest Park	Srisonchai et al. 2023
66	*S.turcosa* Srisonchai & Pimvichai, 2023	OR530087	CUMZ-Zeph0012	Thailand, Loei Province, Mueang Loei District, Phu Pha Lom Forest Park	Srisonchai et al., 2023
67	*S.tujiaphilum* Zhao & Liu, 2022	OP339783	SCAU SD02	China, Guizhou, Tongren City, Jiangkou County, Guanhe Town, Sidu Village	Zhao et al. 2022
68	*S.tujiaphilum* Zhao & Liu, 2022	OP339784	SCAU SD01	China, Guizhou, Tongren City, Jiangkou County, Guanhe Town, Sidu Village	Zhao et al. 2022
69	*S.tujiaphilum* Zhao & Liu, 2022	OP339785	SCAU BHS01	China, Guizhou, Tongren City, Jiangkou County, Taiping Town, Baiheshan Village	Zhao et al. 2022
70	*S.tujiaphilum* Zhao & Liu, 2022	OP339786	SCAU JXT01	China, Guizhou, Tongren City, Jiangkou County, Taiping Town, Jiang-xitun Village	Zhao et al. 2022
71	*S.tujiaphilum* Zhao & Liu, 2022	OP339787	SCAU JXT02	China, Guizhou, Tongren City, Jiangkou County, Taiping Town, Jiang-xitun Village	Zhao et al. 2022
72	*S.tujiaphilum* Zhao & Liu, 2022	OP339788	SCAU SD03	China, Guizhou, Tongren City, Jiangkou County, Guanhe Town, Sidu Village	Zhao et al. 2022
73	*S.tujiaphilum* Zhao & Liu, 2022	OP339789	SCAU DW01	China, Guizhou, Tongren City, Jiangkou County, Dewang Town, Xiaobang Village	Zhao et al. 2022
74	*Arthrosphaerabrandtii* (Humbert, 1865)	FJ409915	FMNH-INS 8650	Tanzania, Usambara hills	Wesener et al. 2010
75	*Cryxusovalis* (Linnaeus, 1758)	JX486069.2	ZFMK MYR0824	Vietnam, Dong Nai Province, Cat Tien National Park	Golovatch et al. 2012
76	*Epicyliosoma* sp.	AF218270	NA	NA	Edgecombe and Giribet 2004
77	*Glomerismarginata* (Villers, 1789)	FJ409909	ZFMK Myr009	Germany, Bonn, Venusberg	Wesener et al. 2010
78	*Prionobeluminthanonense* Donworth & Wesener, 2024	PP297645	MHNG 4E-2	Thailand, Chiang Mai Province, Chom Thong District, Doi Inthanon National Park	Donworth and Wesener 2024
79	*Prionobeluminthanonense* Donworth & Wesener, 2024	PP297646	MHNG 7A	Thailand, Chiang Mai Province, Chom Thong District, Doi Inthanon National Park	Donworth and Wesener 2024
80	*Prionobelumnaevium* Donworth & Wesener, 2024	PP297647	MHNG 4B-1	Thailand, Yala Province, Than To District, Bang Lang National Park, Than To Waterfall	Donworth and Wesener 2024
81	*Prionobelumnaevium* Donworth & Wesener, 2024	PP297648	NHMD 1184671	Thailand, Yala Province, Than To District, Bang Lang National Park	Donworth and Wesener 2024
82	*Prionobelumnaevium* Donworth & Wesener, 2024	PP297649	NHMD 1184672	Thailand, Yala Province, Than To District, Bang Lang National Park	Donworth and Wesener 2024
83	*Prionobelumnaevium* Donworth & Wesener, 2024	PP297650	NHMD 1184673	Thailand, Yala Province, Than To District, Bang Lang National Park	Donworth and Wesener 2024
84	*Sphaeromimussplendidus* Wesener & Sierwald, 2005	FJ409917	FMMC-INS 6702	Madagascar, Sainte Luce S9	Wesener et al. 2010

The inspection of sequencing chromatograms was performed in MEGA 7 (Kumar et al. 2016) in order to check for missing sites. The stop codons and misaligned regions were also carefully checked by aligning all sequences into an appropriate reading frame with a few nucleotide sequences removed at the 5' end and translated to amino acids. All sequences were aligned in MEGA 7 and the aligned data were analysed using JModelTest2 on XSDXE 2.1.6 (Darriba et al. 2012) through the CIPRES Gateway (Miller et al. 2010) to test nucleotide evolution models and to infer the best-fit substitution model for the data.

The phylogenetic trees were generated under two approaches: maximum likelihood (ML) and Bayesian inference (BI). For ML, the analysis was conducted using IQ-tree on XSEDE 1.6.6 (Minh et al. 2020) via the CIPRES Gateway (Miller et al. 2010) with the GTR+I+G model and with 10,000 bootstrap replicates. For BI, we used KAKUSAN 4.0 (Tanabe 2011) to prepare the original file with the adjustments of the best-fit model (GTR+I+G). The analysis was conducted in MrBayes on XSEDE 3.2.7a (Ronquist et al. 2012) via the CIPRES Gateway with Markov Chain Monte Carlo algorithms (MCMC) and with a random starting tree, running for 50 million generations, sampling every 1,000 generations. The ML and BI trees were visualized in FigTree v. 1.4.0 (Rambaut 2010) and then visually processed in Adobe Illustrator 2021. To evaluate node robustness, ML bootstrap support values (BS) > 70% and BI posterior probabilities (PP) > 0.95% are interpreted as strong support (Huelsenbeck and Hillis 1993; Larget and Simon 1999). We also evaluated the intergeneric, interspecific, and intraspecific genetic distances using uncorrected p-distance in MEGA 7 (Kumar et al. 2016) under the pairwise deletion parameter.

## ﻿Results

### ﻿Genetic analyses

The final aligned COI sequences were composed of 658 base pairs, and the sequence annotation contained 375 variable sites, 263 conservative sites and 312 sites were parsimony informative. The percentual distances between outgroups (*Arthrosphaerabrandtii* + *Epicyliosoma* sp. + *Arthrosphaerabrandti* + *Sphaeromimussplendidus*) and *Zephronia* + *Sphaerobelum* + *Prionobelum* + *Cryxus* were 25.11–46.39%. The intergeneric distances between *Zephronia* and *Sphaerobelum* ranged between 7.19 to 31.62%, between 19.13 to 28.63% for *Zephronia* and *Prionobelum*, and between 21.19 to 28.75% for *Zephronia* and *Cryxus*. The interspecific distances within *Zephronia*, *Sphaerobelum*, *Prionobelum* each ranged from 9.91–29.93%, 10.86–31.21% and 20.60–22.12%, respectively. The intraspecific distances of *Z.chantaburiensis* sp. nov. and *Z.macula* sp. nov. ranged between 0–0.15% and 0.15–4.40%, respectively. The two new species were separated from each other by an interspecific p-distance of 18.94–19.71%. Information regarding genetic distances is presented in the Suppl. material 1.

**Figure 1.**
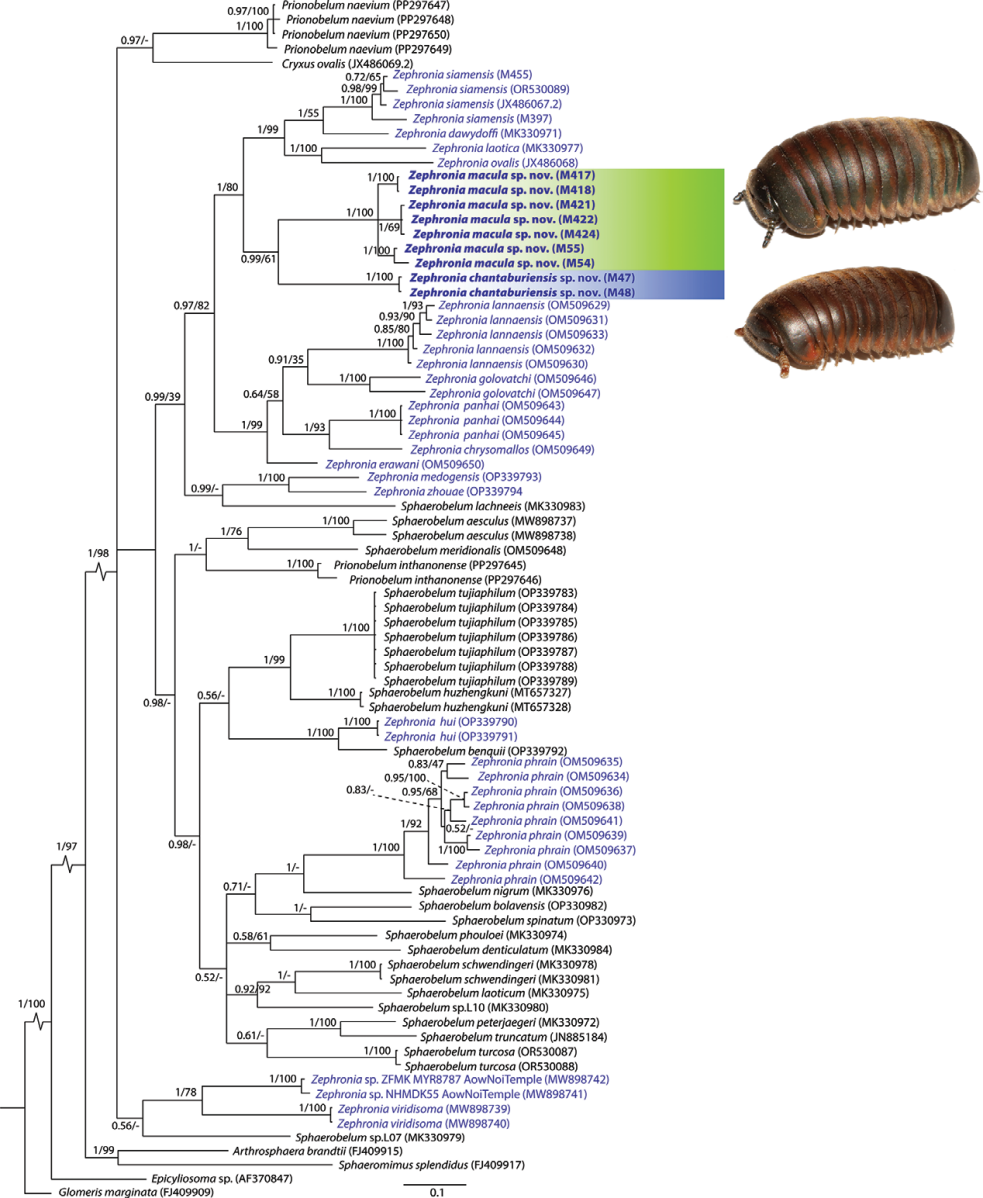
Phylogenetic tree based on Bayesian Inferences (BI). Numbers at nodes indicate Bayesian posterior probabilities (PP)/ bootstrap support (BS). The dash “-” at the nodes refer to a different topology in the BI analysis compared to ML (see Supplemental Material 2). Colours denote the two new species (*Zephroniachantaburiensis* sp. nov. and *Zephroniamacula* sp. nov.), corresponding to the live photograph. Scale bar represents substitutions/site.

BI and ML methods returned different topologies, especially at deep nodes (Fig. 1 and Suppl. material 2). However, the relationships at younger internal nodes and terminal nodes of most species were still similar to each other, especially for *Zephronia* sensu stricto (s.s.). The tree topology based on BI compared to ML is presented as an overview to interpret the results (Fig. 1).

The low support values of deep nodes in our trees based on the COI gene alone do not allow for a conclusion on the relationships among the species of *Zephronia* as well as of those in *Sphaerobelum*, *Prionobelum*, and *Cryxus*. The preliminary data, however, questions the monophyly of these genera.

According to the results, the trees from both BI and ML clearly show that the genus *Zephronia* is recovered as polyphyletic, with the clades formed by *Z.viridisoma* and *Zephronia* sp. (Aow Noi Temple) as a well-supported sister group to all other species of *Zephronia* and *Sphaerobelum* located in a trichotomy with a branch supporting *Prionobelum* and *Cryxus* Leach, 1814 (PP = 1.0, BS = 98). *Z.hui* and *Z.phrain* cluster among species of *Sphaerobelum*, but with low statistical support. Morphologically aberrant species of *Sphaerobelum* cluster with *Prionobeluminthanonense*. All other Thai, Laotian and Cambodian *Zephronia* s.s. are in a well-supported monophylum in both BI and ML (PP = 0.97, BS = 82) including the two new species described here (Fig. 1). Regarding the new species, each is recovered as monophyletic with strong support (BS = 100, PP = 1.0) and the two are grouped together with members of the *Zephronia* s.s. species group (*Z.dawydoffi*, *Z.laotica*, *Z.ovalis*, and *Z.siamensis*).

### ﻿Systematics

**Family Zephroniidae Gray, 1843**

**Subfamily Zephroniinae Gray, 1843**

**Tribe Zephroniini Gray, 1843**

#### ﻿Genus Zephronia

Taxon classificationAnimaliaSphaerotheriidaZephroniidae

Gray, 1832

8B321F4E-2E9F-58A9-9B3D-07B9612F036A

##### Differential diagnosis.

Differs from all other genera of Zephroniinae by the combination of the following characters: 1) Body length 18–50 mm. 2) Antennae flattened laterally, usually axe-shaped, with numerous (> 4) apical cones. 3) Endotergum (tergite underside) weakly modified: posterior margin (pm) usually flat; outer area (os) without setae; marginal bristles arranged in 1–5 rows; middle area (ma) often with a single row of circular cuticular impressions. 4) Tarsi of legs 5–21 often with more than one apical spine (with very few species with only 1) and several ventral spines. 5) Anterior telopods with four podomeres distal to syncoxite; telopoditomere 2 with a large, curved process forming a clamp-like; telopoditomeres 3 and 4 simple. 6) Posterior telopods with four telopoditomeres; immovable finger (process of telopoditomere 2 slender, apically curved; telopoditomeres 3 and 4 with two membranous lobes; telopoditomere 3 also with a row of conspicuous, crenulated teeth, larger than elopoditomere 4; telopoditomere 4 with few (one or two) sclerotized spines. 7) Female vulvae conspicuous, slender; operculum usually round.

*Zephronia* is one of the most species-rich genera of the family with more than 50 species currently placed in the genus. Numerous species currently placed in the genus are in need of a revision, some only known from the female, and might be placed in separate genera in the future. The posterior telopod in *Zephronia* consists of four podomeres, unlike *Castanotherium* Pocock, 1895 whose species have three podomeres only. Podomere 4 of the posterior telopod in *Zephronia* is not strongly curved or overlapping the process of podomere 2 as in *Cryxus* Leach, 1814. The process of podomere 2 in *Zephronia* is never apically enlarged or swollen like in *Sphaerobelum* Verhoeff, 1924. The osterior telopod on podomere 3 in *Zephronia* has crenulated teeth, unlike *Kophosphaera* Attems, 1935 which lacks sclerotized teeth or spines entirely. The posterior telopod of *Zephronia* species is identical to those of species of *Sphaeropoeus* Brandt, 1833, *Prionobelum* Verhoeff, 1924, *Tigridosphaera* Jeekel, 2000, and *Indosphaera* Attems, 1935. *Zephronia* species differ from species of *Sphaeropoeus* and *Prionobelum* in the anterior telopods, lacking the characteristic processes on joints three (*Sphaeropoeus*) and four (*Prionobelum*). *Zephronia* species differ from *Indosphaera* in the coxae of leg 2 in females being separate, not fused. *Zephronia* species are currently impossible to distinguish from species of *Tigridosphaera* as that species-poor genus is in need of revision. Females of *Zephronia* differ in their shape of the operculum of the vulva from species of *Indosphaera*, where it is much lower than in *Zephronia* species, as well as from species of *Sphaeropoeus* and *Prionobelum* where the operculum is greatly enlarged and almost rectangular in shape.

###### ﻿*Zephronia* s.s.

Differing from all *Zephronia* s.l. by the position of the organ of Tömösváry, which is located at the brim and not inside the antennal groove as in all other known Sphaerotheriida. *Zephronia* s.s. includes the type species of the genus, *Z.ovalis*, as well as *Z.chantaburiensis* sp. nov., *Z.chrysomallos*, *Z.dawydoffi*, *Z.erawani*, *Z.enghoffi*, *Z.golovatchi*, *Z.hui* Liu & Wesener, 2022, *Z.konkakinhensis* Semenyuk, Golovatch & Wesener, 2018, *Z.lannaensis*, *Z.laotica*, *Z.macula* sp. nov., *Z.medongensis* Zhao & Liu, 2022, *Z.montis* Semenyuk, Golovatch & Wesener, 2018, *Z.panhai*, *Z.siamensis* and *Z.zhouae* Zhao & Liu, 2022. See more details for the genus in Wesener (2016) and Semenyuk et al. (2018), and a recent update of Thai species in Bhansali and Wesener (2022).

### ﻿List of 11 *Zephronia* species occurring in Thailand

1. *Z.chantaburiensis* Srisonchai & Wesener, sp. nov.
2. *Z.chrysomallos* Bhansali & Wesener, 2022
3. *Z.enghoffi* Srisonchai & Likhitrakarn, 2021
4. *Z.erawani* Bhansali & Wesener, 2022
5. *Z.golovatchi* Srisonchai & Likhitrakarn, 2021
6. *Z.lannaensis* Likhitrakarn & Golovatch, 2021
7. *Z.macula* Srisonchai & Wesener, sp. nov.
8. *Z.panhai* Srisonchai & Likhitrakarn, 2021
9. *Z.phrain* Likhitrakarn & Golovatch, 2021
10. *Z.siamensis* Hirst, 1907
11. *Z.viridisoma* Rosenmejer & Wesener, 2021

#### ﻿ Zephronia chantaburiensis

Taxon classificationAnimaliaSphaerotheriidaZephroniidae

Srisonchai & Wesener sp. nov.

62D650CF-2756-59DE-8674-78554ACBEF27

https://zoobank.org/FEEDDAE0-362F-4368-BFBF-A2238866B8F0

Figs 2 , 3 , 4 , 5 , 6 , 7 , 14A–D , 15

##### Type specimens:

***Holotype*** • ♂ (CUMZ-MYR0013); Thailand, Chantaburi Province, Tha Mai District, Wat Khao Sukim (Khao Sukim Temple); 12°45'47"N, 102°01'56"E; ca 53 m a.s.l.; 14 June 2023; leg. R. Srisonchai and KKUMZ students. ***Paratypes*.** • 29 ♂, 16 ♀ (CUMZ-MYR0014), same data as holotype; • 2 ♂, 1 ♀ (NHMD1184695), same data as holotype; • 1 ♂, 1 ♀ (NHMW10436), same data as holotype; • 1 ♂, 1 ♀ (ZFMK-MYR13659), same data as holotype.

##### Additional material.

• 26 juveniles (CUMZ), same data as holotype.

##### Diagnosis.

**Figure 2.**
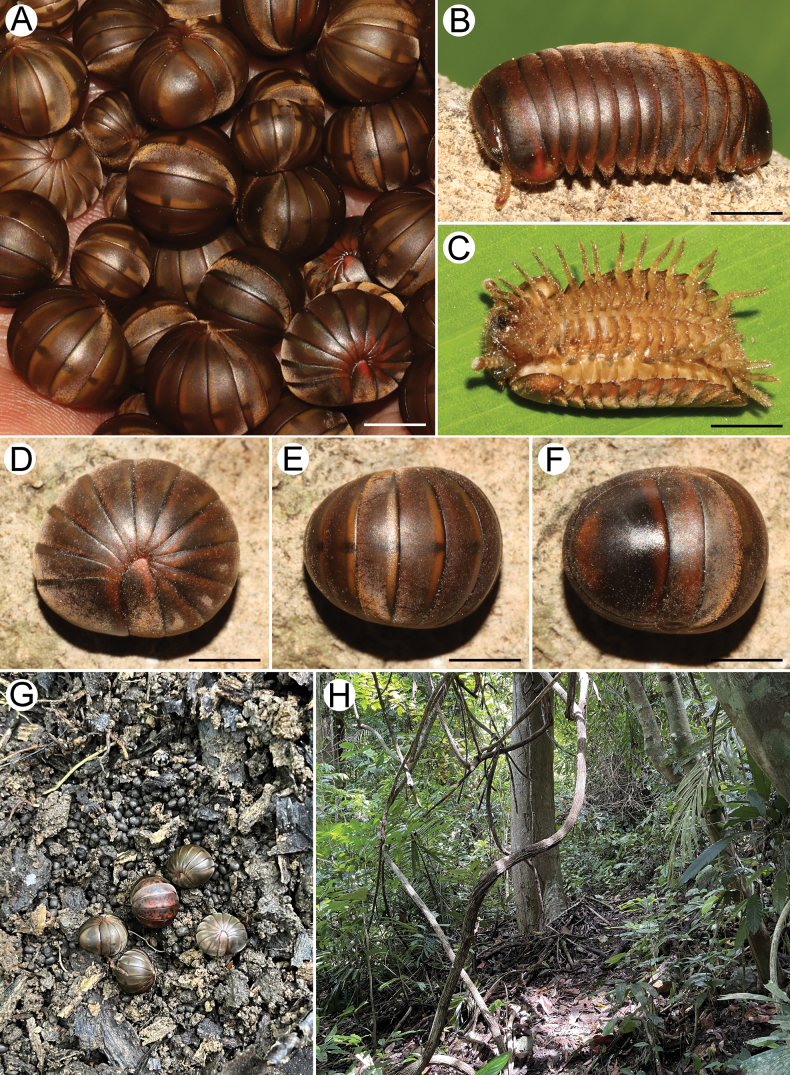
Photograph of live specimens of *Zephroniachantaburiensis* sp. nov. and habitats **A–F** paratypes (CUMZ-MYR0014) **G** coexisting species (*Z.macula* sp. nov.) **H** granite habitat at the type locality. Scale bars: 0.5 mm.

**Figure 3.**
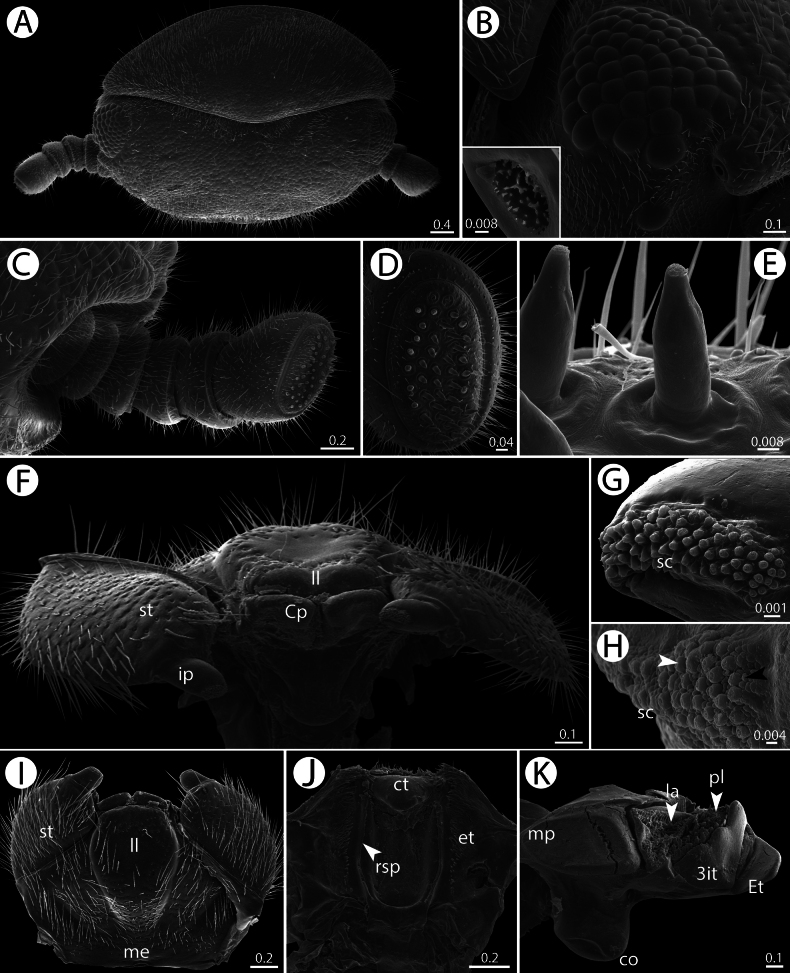
*Zephroniachantaburiensis* sp. nov., ♂ paratype (CUMZ-MYR0014) — SEM**A** head, collum and antenna, dorsal view **B** ommatidia, inset: organ of Tömösváry **C** antenna, anterior view **D** antennal disc, anterior view **E** apical cones, lateral view **F, I** gnathochilarium, posterior and ventral views, respectively **G** sensory cones on central pad, ventral view **H** sensory cones on inner palpi, black and white arrows point to different types of cones, ventral view **J** epipharynx, ventral view **K** mandible, mesal view. Abbreviations: 3it = 3-combed inner tooth, co = condylus, Cp = central pad, ct = central tooth, et = external tooth, Et = external tooth, Ia = inner area, il = incisura lateralis, ip = inner palpi, ll = lamellae linguales, me = mentum, mp = molar plate, pl = pectinate lamellae, rsp = row of spines, sc = sensory cone, st = stipite. Scale bars in millimeters.

The position of the organ of Tömösváry at the brim and not inside the antennal groove (Fig. 3B) identifies this species as a member of the *Zephronia* s.s. species group (see Semenyuk et al. 2018), with which it also aligns genetically (Fig. 1). This small brown species (body length ca 20 mm) with short golden hair (Fig. 2A–F) differs from all other *Zephronia* s.s. species, except for *Z.macula* sp. nov. found in direct sympatry, described below, in the presence of only a single apical spine on the tarsus of legs 4–21 (2 or 3 in the other species). Midbody endotergum with one row of marginal bristles with longest bristles reaching up to posterior margin, immovable finger (process) of telopoditomere 2 on anterior telopod relatively long and slender. Similar in these respects only to *Z.macula* sp. nov. but differs from the latter species by the tergite coloration lacking dark or greenish-dark colour spots, the operculum of the female being more slender, the femur of the walking legs being slightly wider than long (slightly longer than wide in *Z.macula* sp. nov.), and the female subanal plate having a strongly concave margin. Genetically distant from other species by 18.94–26.82% p-distance in the COI barcoding fragment.

##### Description.

***Measurements***: Male holotype. Body length 20 mm. Width, of thoracic shield 9 mm, of tergite 7 = 10 mm (= broadest). Height of tergite 7 = 7 mm (= highest). Males: body length = 19–23 mm. Width, of thoracic shield = 8–9 mm, of tergite 7 = 9–10 mm. Height of tergite 7 = 6–8 mm. Females: body length = 19–22 mm. Width, of thoracic shield = 8–9 mm, of tergite 7 = 9–11 mm. Height of tergite 7 = 6–8 mm (= highest).

***Colouration*** (Fig. 2A–F): In life with body of brown colour. Head, collum, thoracic shield and tergites brown. Antenna, legs, and venter light brown. Anal shield dark brown (rarely brown), except anterior and posterior margins pale brown. Tergites dorsally with dark brown stripe, clearly seen when rolled up. Specimens in alcohol after one year changed to pale brown.

***Head*** (Fig. 3A, B): Trapeziform, densely setose; anterior part with setae longer than in posterior part; each seta located inside small pit. With 57–65 ommatidia (ocelli) in males and 60–65 in females. Aberrant ommatidium located at brim of antennal groove. Organ of Tömösváry situated near the base of antenna, separated from eye field. No sclerotized crest/ridge between antennal socket and eye field.

***Antennae*** (Figs 2B, C, 3A, C–E): Short and stout, covered by long and dense setae; last antennomere reaching back to leg pair 2 or 3. Lengths of antennomeres: 1=2=3=4<5<<6. Antennomere 6 slightly flattened apically, axe-shaped; apically with sensilla basiconica. Apical disc flat; with 30–41 (male) or 25–35 (female) apical cones.

***Epipharynx*** (Fig. 3J): With a regular central tooth (ct); inner tooth conspicuous and flat; laterally with numerous long external teeth (et); inner area with a single row of fringed spines (rsp) on each side.

***Gnathochilarium*** (Fig. 3F–I): Structure as typical as for Sphaerotheriida. Lamellae linguales (ll) oval, slightly concave apically, with long setae. Central pads (Cp) modified, with numerous “pillows” of sensory cones (sc) (Fig. 3H); two types of sensory cones (one with a pillow and another without a pillow). Stipites (st) large, densely setose; located laterally to lamellae linguales. Mentum (me) large, fused, with sparse and long setae. Lateral palpi inconspicuous. Inner palpi (ip) with sensory cones (sc) arranged in single field (Fig. 3G).

***Mandibles* (*gnathal lobe*)** (Fig. 3K): With undivided external tooth (Et) and with conspicuous 3-combed inner tooth (3it). With 5–7 pectinate lamellae (pl). Inner area (Ia) with group of long and tiny teeth. Molar plate (mp) flat, velvet-like; lacking a membranous fringe. Condylus (co) conspicuous, apically with one distinct ridge.

**Figure 4.**
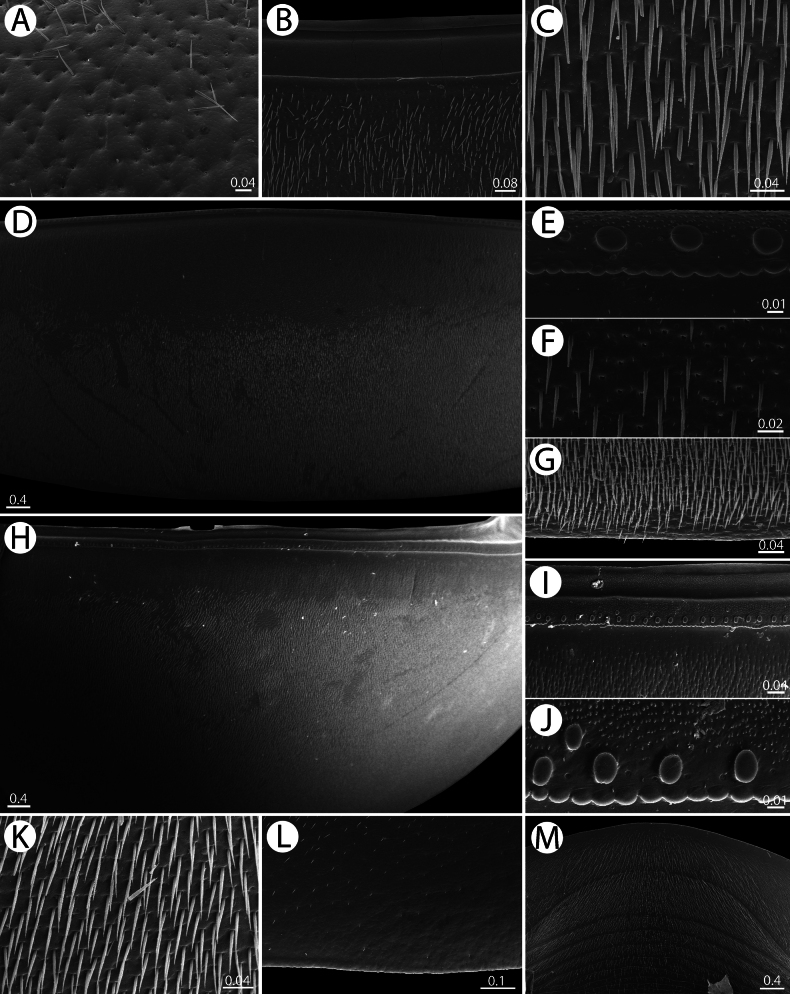
*Zephroniachantaburiensis* sp. nov., ♂ paratype (CUMZ-MYR0014) — SEM**A** surface of collum **B, C** thoracic shield, all in dorsal views (anterior margin and surface, respectively) **D–G** midbody tergite, all in dorsal views (overview, anterior margin, surface, and posterior margin, respectively) **H–J** anal shield, all in dorsal views (overview, anterior margin and cuticular impressions, respectively) **K–M** anal shield, underside, all in ventral views (surface, posterior margin, and overview, respectively). Scale bars in millimetres.

***Tegument*** (Figs 2A–F, 4): Quite dull; collum, thoracic shield, tergite and anal shield densely covered by tiny golden setae; each seta located in a pit. Anterior margins of midbody tergite and of anal shield with lower number of setae than posterior margins. Posterior rim in dorsal and ventral side of anal shield with a few small setae.

***Collum*** (Figs 2B, 3A): Subsemicircular; tip of lateral margin obtuse, densely setose.

***Thoracic shield*** (Fig. 4B): With shallow and large groove separated by a large and long ridge. Slope towards groove without keel. Groove and ridge smooth, without setae.

***Midbody tergite*** (Fig. 4D–G): With a row of oval impressions and numerous tiny tubercles at anterior edge. Inner area with crenate barrier, forming a wide and shallow groove. Tips of midbody paratergites projecting caudoventrad.

***Anal shield*** (Fig. 4H–M): Slightly sexually dimorphic, in female large and well-rounded, in male slightly slenderer. With a row of oval impressions and several tiny tubercles at anterior edge. Inner area with crenate barrier, forming a wide and shallow groove. Underside with a single, short, black locking carina; twice as long as those of tergites, as long as half of tarsus.

***Endotergum of thoracic shield and midbody tergite*** (Fig. 14A–D): Thoracic shield similar to midbody tergite. Posterior margin (pm) flat, regular. Outer area (os) without setae. Marginal bristles arranged in one row, tip of the longest bristles very slightly protruding above posterior margin. Middle area (ma) with a single row of conspicuous, circular cuticular impressions; distance between impressions twice as long as individual diameter. Inner area (ia) without tubercles or setae.

**Figure 5.**
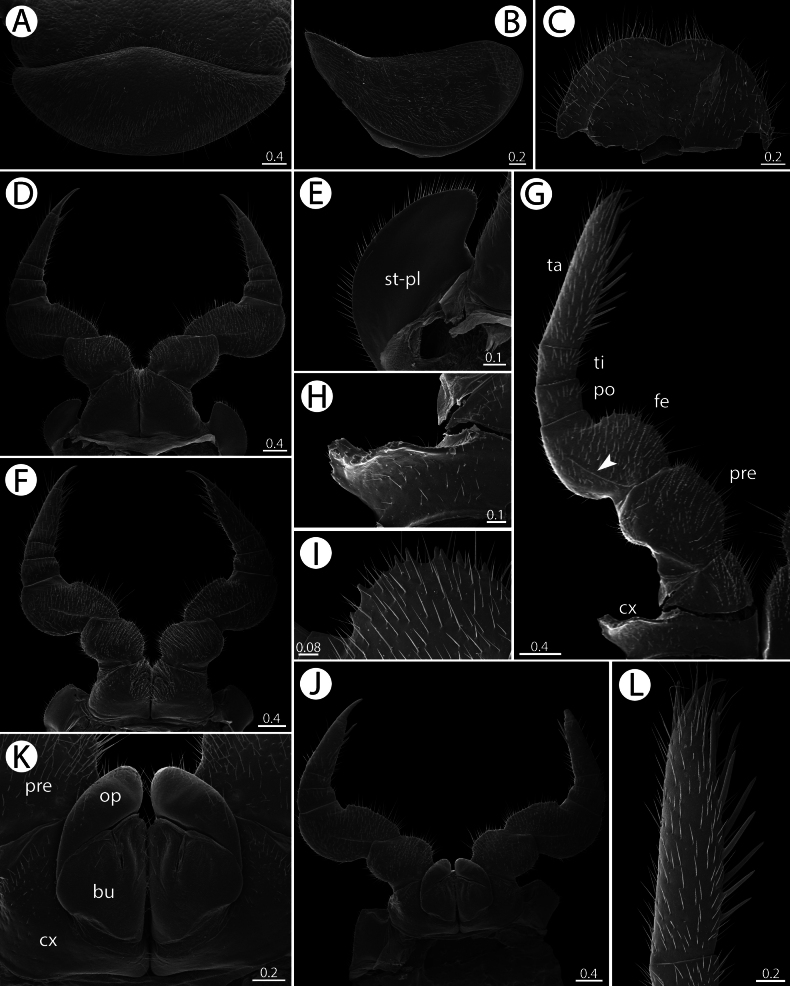
*Zephroniachantaburiensis* sp. nov. SEM. **A–G, L** ♂ paratype (CUMZ-MYR0014) **J, K** ♀ paratype (CUMZ-MYR0014) **A** collum **B** first pleurite **C** subanal plate **D** legs 1 **E** stigmatic plate 1 **F** legs 2 with gonopores **G** leg 10 (arrow points to femoral ridge) **H** coxal process on leg 10 **I** small teeth on femur **J** leg 2 with vulvae **K** vulvae **L** tarsus 10. Abbreviation: bu = bursa, cx = coxa, fe = femur, op = operculum, po = postfemur, pre = prefemur, st-pl = stigmatic plate, ta = tarsus, ti = tibia. Scale bars in millimetres.

***Pleurite (laterotergite)*** (Fig. 5B): With short setae. First pleurite slender, boomerang-shaped; apical margin slightly attenuated; strongly projecting into a wide tip. Pleurite 2 wider than first one, projecting into obtuse tip. The remaining pleurites flat and wide, lamella-like, apical margin slightly extended.

***Subanal plate of female*** (Fig. 5C): Large and wide, semicircular; sparsely setose; apical margin strongly concave.

***Stigmatic plates*** (Fig. 5D, E): First stigmatic plate slender, apex well-rounded, slightly curving towards coxa (Fig. 5E). Second stigmatic plate in both sexes without any curve (Fig. 5F, J).

***Legs*** (Fig. 5D–L): All podomeres densely setose. Coxa (cx) large; coxal process absent in first and second legs; legs 3–21 marginally with large and dentate process. Prefemur (pre) short and stout; apico-mesally with a weak projection; mesal margin with small spines. Femur (fe) stout; with long ridge in all leg-pairs; apico-mesally massively enlarged with a strong projection carrying dentate margin (conspicuous teeth on margin). Postfemur (po) and tibia (ti) short. Tarsus (ta) of midbody legs quite long and slender; as long as length of prefemur+femur combined; first two leg-pairs without an apical spine; leg-pair 1 with 1 ventral spine; leg-pair 2 with 3 ventral spines; leg-pair 3 with 5 or 6 ventral spines and 1 apical spine; leg pairs 4 with 7 or 8 ventral spines and 1 apical spine; leg pairs 5–21 with 7–9 ventral spines and 1 apical spine. In leg 9, femur slightly wider than long (1.1×), tarsus 4× longer than wide. Claw normal, with a small notch at base.

***Male sexual characters*** (Fig. 5F): Gonopore large, covered by long setae; with divided sclerotized plates, triangular.

**Figure 6.**
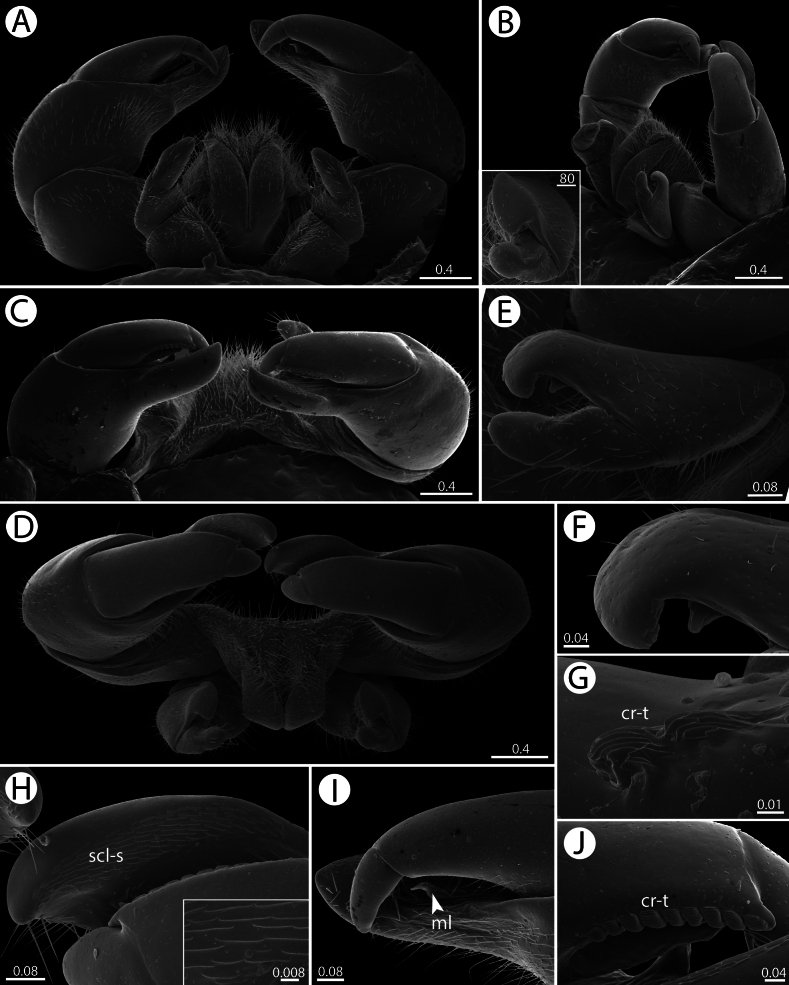
*Zephroniachantaburiensis* sp. nov., ♂ paratype (CUMZ-MYR0014) — SEM**A–D** overview of telopods (anterior view, sublateral view with emphasis on anterior telopod, posterior view and ventral view, respectively) **E–G** anterior telopod (telopoditomeres 2–4, immovable finger and crenulated teeth, respectively) **H–J** posterior telopod, all in sublateral views (sclerotize spots on immovable finger, telopoditomeres 2–4 with emphasis on membranous lobe and crenulated teeth, respectively). Abbreviations: cr-t = crenulated teeth, ml = membranous lobe, scl-s = sclerotized spots. Scale bars in millimeters.

***Anterior telopods*** (Fig. 6A, B, E–G): First telopoditomere rectangular. Telopoditomere 2 large, as long as telopoditomere 3. Immovable finger (process of telopoditomere 2) long and slender; almost as long as movable finger (= combination of telopoditomeres 3+4); clearly seen in posterior and anterior views; strongly curved; tip obtuse, directed anteriad; with a membranous lobe and sclerotized spots located at inner margin. Telopoditomere 3 as long as telopoditomere 4, clearly demarcated from telopoditomere 4 by conspicuous suture; apically with few crenulated teeth (cr-t). Tepoloditomere 4 apically with large and long setae.

**Figure 7.**
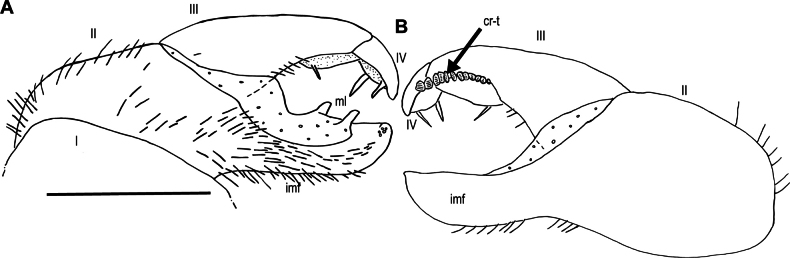
*Zephroniachantaburiensis* sp. nov., ♂ paratype (ZFMK-MYR13659), drawings — right posterior telopod **A** anterior view **B** posterior view. Abbreviations: cr-t = crenulated teeth, imf = immovable finger, ml = membranous lobe. Scale bar: 0.5 millimeters micrometres.

***Posterior telopods*** (Figs 6A–D, H–J; 7): Consisting of 4 telopoditomeres. First telopoditomere rectangular, slightly longer than wide. Telopoditomere 2 large and stout. Immovable finger (process of telopoditomere 2) long and slender, 3× longer than wide, with a characteristic swelling mesally; slightly longer than movable finger (= combination of telopoditomeres 3+4); attenuate near tip; tip obtuse; tip in situ curving anteriad; inner margin with few conspicuous sclerotized spots (scl-s) and two membranous lobes (ml). Telopoditomere 3 long, 2.5× longer than wide, with a membranous ledge and single spine at excavate inner margin. Telopoditomere 4 relatively short; 4× shorter than telopoditomere 3; 1.5× longer than wide, slightly tapering toward apex; with two long spines located on membranous ledge; posteriorly with 9–12 crenulated teeth (cr-t). Telopoditomeres 1 and 2 in anterior view mostly covered by setae, in posterior view mostly glabrous. Telopoditomeres 3 and 4 glabrous, except small area at basal part of telopoditomere 3 with setae. Inner horns with sharp-edged tips, slightly curved caudad.

***Female sexual characters*** (Fig. 5J, K): Vulva large, slender, sparsely setose; covering almost 1/2 of coxa; located at mesal margin; extending mesally to base of prefemur. Operculum (op) round, mesal margin protruding into an oval-like lobe, tip of operculum obtuse. Bursa (bu) large, demarcated from operculum by a triangular groove.

##### Remarks.

Negligible colour variation is observed in the anal shield between different living specimens. Generally, they are dark brown in most specimens and pale brown in others. Females are typically of the same size as males.

##### Distribution and habitat.

*Zephroniachantaburiensis* sp. nov. is currently known only from the type locality in Chantaburi Province and is therefore here regarded as an endemic species. The new species was encountered during the day time in evergreen forest in granitic habitat (Fig. 2H). They hide themselves beneath thick leaf litter and decayed wood logs (Fig. 2G). Co-occurring and dominant millipedes are included, *Z.macula* sp. nov. and *Desmoxyteseuros* Srisonchai et al., 2018.

##### Etymology.

The name is an adjective referring to the province (Chantaburi) where the type locality is located.

#### ﻿ Zephronia macula

Taxon classificationAnimaliaSphaerotheriidaZephroniidae

Srisonchai & Wesener sp. nov.

F69B1A1D-C0F2-5433-8EC1-CF3319469C6A

https://zoobank.org/84892BB6-C242-4C03-8639-39F8A0D97683

Figs 8 , 9 , 10 , 11 , 12 , 13 , 14E–H , 15

##### Type specimen.

***Holotype*** • ♂ (CUMZ-MYR0015); Thailand, Chantaburi Province, Kaeng Hang Maeo District, near Khao Wong Kot Cave, Thamma Sooksawang Temple; 12°53'51.7"N, 101°48'59.7"E; ca. 60 m a.s.l.; 14 June 2023; leg. R. Srisonchai and KKUMZ students. ***Paratypes*.** • 53 ♂, 74 ♀ (CUMZ-MYR0016), same data as holotype; • 2 ♂, 2 ♀ (NHMD1184696), same data as holotype; • 2 ♂, 2 ♀ (NHMW10437), same data as holotype; • 2 ♂, 2 ♀ (ZFMK-MYR13660), same data as holotype.

##### Additional material.

• 29 juveniles (CUMZ); Thailand, Chantaburi Province, Kaeng Hang Maeo District, near Khao Wong Kot Cave, Thamma Sooksawang Temple; 12°53'51.7"N, 101°48'59.7"E; ca. 60 m a.s.l.; 14 June 2023; leg. R. Srisonchai and KKUMZ students; • 26 ♂, 31 ♀ (CUMZ); Thailand, Chantaburi Province, Tha Mai District, Wat Khao Sukim (Khao Sukim Temple); 12°45'47"N, 102°01'56"E; ca. 53 m a.s.l.; 14 June 2023; leg. R. Srisonchai and KKUMZ students; • 18 ♂, 26 ♀ (CUMZ); Thailand, Chantaburi Province, Khlung District, Thaeo Klong Khlung Monastery; 12°28'53.3"N, 102°13'06.3"E; ca. 74 m a.s.l.; 14 June 2023; leg. R. Srisonchai and KKUMZ students; • 26 ♂, 65 ♀; 47 juveniles (CUMZ); Thailand, Chantaburi Province, Makham District, Wat Khao Banchob, 12°51'09.0"N, 102°12'15.0"E; ca. 110 m a.s.l.; 13 June 2023; leg. R. Srisonchai and KKUMZ students • 33 ♂, 43 ♀ (CUMZ); Thailand, Chonburi Province, Bo Thong District, Wat Khao Yai Aran Khiri; 13°14'54.7"N, 101°37'29.6"E; ca. 160 m a.s.l.; 15 June 2023; leg. R. Srisonchai and KKUMZ students; • 66 ♂, 25 ♀ (CUMZ); Thailand, Rayong Province, Makham District, Wat Pa Theprangsi (= Wat Khao Hin Tang); 12°42'03.3"N, 101°32'17.3"E; ca. 80 m a.s.l.; 14 June 2023; leg. R. Srisonchai and KKUMZ students; • 5 ♂, 7 juveniles (CUMZ); Thailand, Sa Kaeo Province, Khlong Hat District, Phet Pho Thong Cave; 13°24'53.5"N, 102°19'35.1"E; ca. 245 m a.s.l.; 12 June 2023; leg. R. Srisonchai and KKUMZ students; • 1 ♂, 2 ♀ (CUMZ); Thailand, Sra Kaeo Province, Mueang Sra Kaeo District, Wat Tham Khao Maka; 13°47'11.9"N, 101°56'51.8"E; ca. 70 m a.s.l.; August 2014; leg. ASRU members

##### Diagnosis.

The position of the organ of Tömösváry in this small *Zephronia* with an axe-shaped antennomere 6 identifies *Z.macula* sp. nov. as a member of the *Zephronia* s.s. species-group (Semenyuk et al. 2018). *Z.macula* sp. nov. differs from all other known species of the group except for the syntopic *Z.chantaburiensis* sp. nov., in the presence of only a single apical spine on the tarsi of legs 4–21 (at least 2 or 3 in the other *Zephronia*). *Z.macula* sp. nov. differs from *Z.chantaburiensis* sp. nov. by having a combination of distinct characters, viz. tergite with dark or greenish dark spots, femur of walking legs less strongly widened, slightly longer than wide (wider than long in *Z.chantaburiensis* sp. nov.), bristles of midbody endotergum reaching to posterior margin, operculum of female vulva not protruding and round, and immovable finger (process) of telopoditomere 2 of the anterior telopod more curved and much longer. Genetically distant from others by a p-distance of the COI barcoding fragment of 17.93–25.13%.

##### Description.

***Measurements***: Male holotype. Body length 18 mm. Width, of thoracic shield 8 mm, of tergite 7 = 9 mm (= broadest). Height of tergite 7 = 6 mm (= highest). Males: body length = 18–20 mm. Width, of thoracic shield = 8–9 mm, of tergite 7 = 8–10 mm. Height of tergite 7 = 6–7 mm. Females: body length = 18–20 mm. Width, of thoracic shield = 8–9 mm, of tergite 7 = 9–10 mm. Height of tergite 7 = 6–8 mm (= highest).

**Figure 8.**
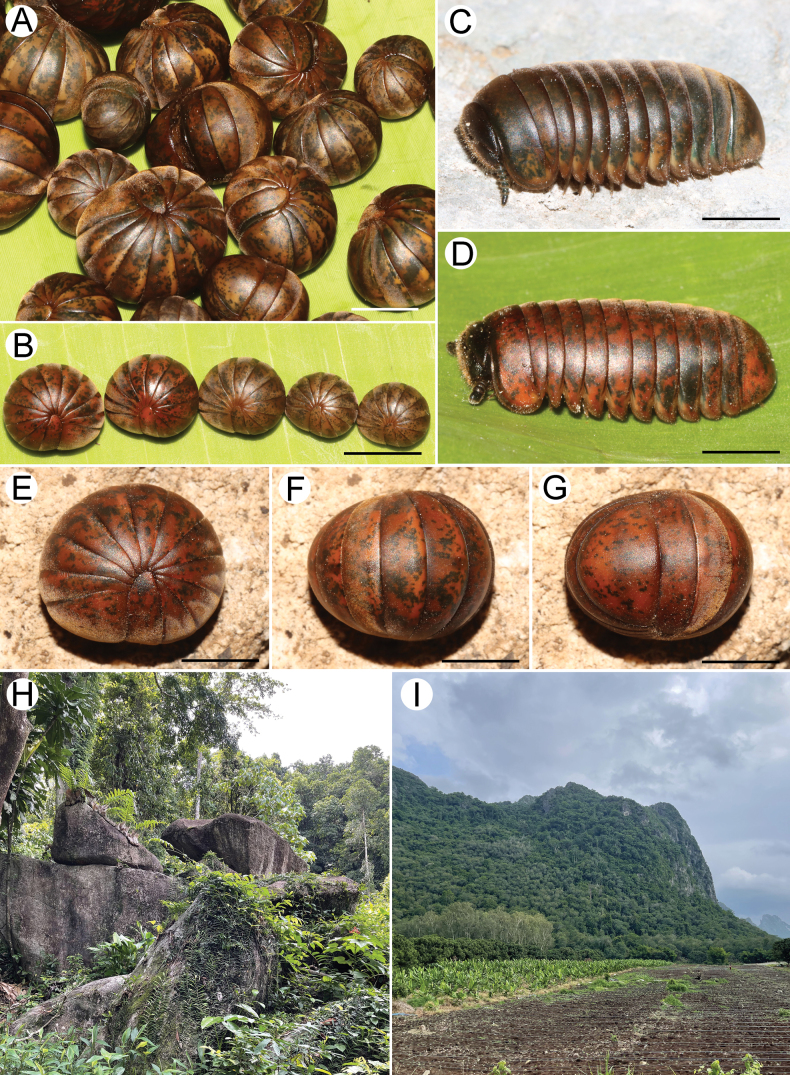
Photograph of live specimens of *Zephroniamacula* sp. nov. and habitats **A–G** paratypes (CUMZ-MYR00016) **H** habitat at Thaeo Khlong Khlung monastery (granitic rocks) **I** habitat at Phet Pho Thong cave (limestone). Scale bars: 0.5 mm.

***Colouration*** (Fig. 8A–G): Specimen in life with body of brown/greenish brown/reddish brown colour, tergite with dark or greenish dark spots. Head, antenna, and collum dark brown. Thoracic shield, tergites and anal shield brown. Colour in alcohol after one year faded to brown.

**Figure 9.**
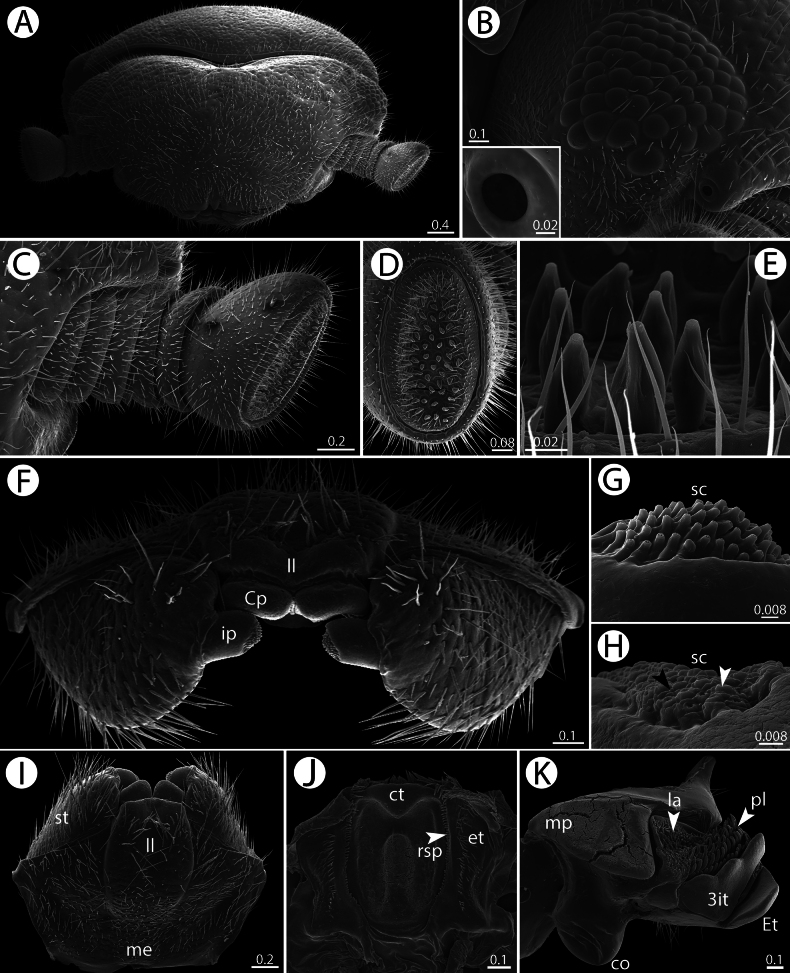
*Zephroniamacula* sp. nov., ♂ paratype (CUMZ-MYR0016) — SEM**A** head, collum and antenna, dorsal view **B** ommatidia, inset: organ of Tömösváry **C** antenna, anterior view **D** antennal disc, anterior view **E** apical cones, lateral view **F, I** gnathochilarium, posterior and ventral views, respectively **G** sensory cones on central pad, ventral view **H** sensory cones on inner palpi, black and white arrows point to different types of cones, ventral view **J** epipharynx, ventral view, **K** mandible, mesal view. Abbreviations: 3it = 3-combed inner tooth, co = condylus, Cp = central pad, ct = central tooth, et = external tooth, Et = external tooth, Ia = inner area, il = incisura lateralis, ip = inner palpi, ll = lamellae linguales, me = mentum, mp = molar plate, pl = pectinate lamellae, rsp = row of spines, sc = sensory cone, st = stipites. Scale bars in millimetres.

***Head*** (Fig. 9A, B): Trapeziform, densely setose; each seta located inside small pit. With 55–65 ommatidia (ocelli) in males and 61–70 in females. At rim of antennal groove with aberrant ommatidium. Organ of Tömösváry located near base of antenna, clearly separated from eye field. No sclerotized crest/ridge between antennal socket and eye field.

***Antennae*** (Figs 8C, D, 9A, C–E): Short and stout, covered by long and dense setae; last antennomere reaching back to leg pair 2 or 3 when stretched ventrally. Lengths of antennomeres: 1=2=3=4<5<<6. Antennomere 6 strongly flattened apically, axe-shaped; apically with sensilla basiconica. Apical disc slightly concave, with 48–57 apical cones (male) or 36–43 (female).

***Epipharynx*** (Fig. 9J): With a large central tooth (ct); inner tooth conspicuous and swollen; laterally with group of long external teeth (et); inner area with a single row of fringed spines (rsp) on each side.

***Gnathochilarium*** (Fig. 9F–I): As in *Z.chantaburiensis* sp. nov. Lamellae linguales (ll) oval, apically concave, with long setae. Central pads (Cp) modified, with numerous “pillows” of sensory cones (sc) (Fig. 9H); two different types of sensory cones (one with a pillow and another without a pillow). Stipites (st) large and stout, densely setose. Mentum (me) large and broad, fused, with long setae. Lateral palpi inconspicuous. Inner palpi (ip) with sensory cones (sc) arranged in single field (Fig. 9G).

***Mandibles* (*gnathal lobe*)** (Fig. 9K): With undivided external tooth (Et) and with prominent 3-combed inner tooth (3it). With 5–6 pectinate lamellae (pl). Inner area (Ia) with group of long and tiny teeth, spine-like. Molar plate (mp) flat, velvet-like; lacking a membranous fringe. Condylus (co) conspicuous, apically with 2 distinct ridges.

**Figure 10.**
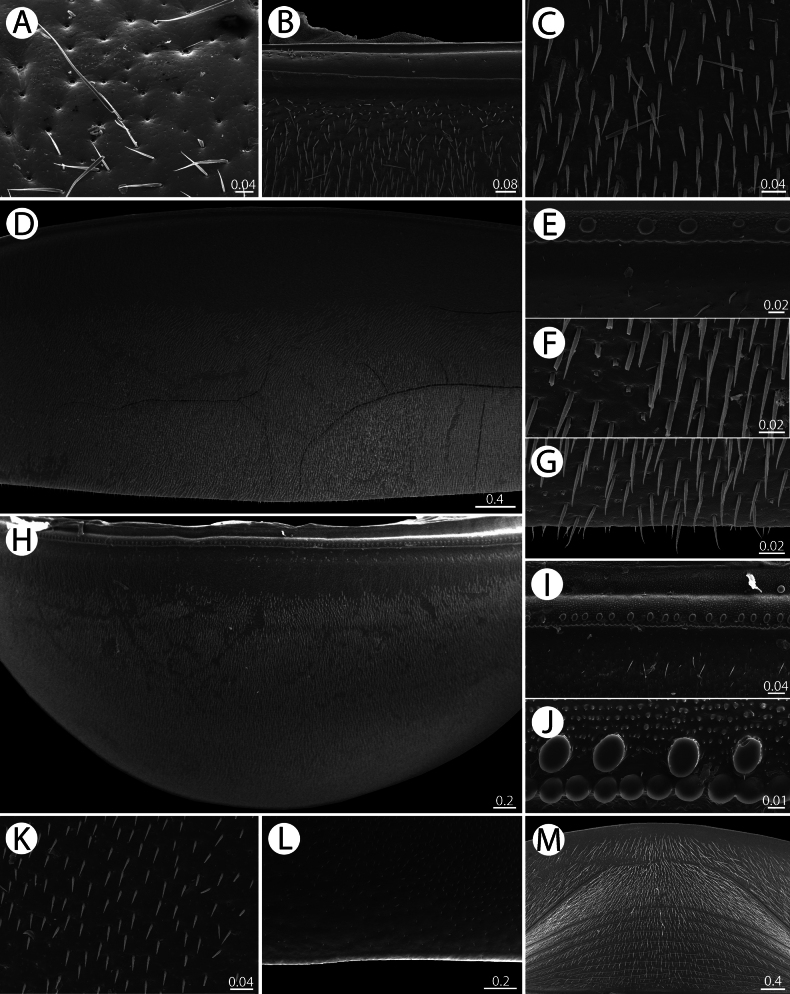
*Zephroniamacula* sp. nov., ♂ paratype (CUMZ-MYR0016) — SEM**A** surface of collum **B, C** thoracic shield, all in dorsal views (anterior margin and surface, respectively) **D–G** midbody tergite, all in dorsal views (overview, anterior margin, surface, and posterior margin, respectively) **H–J** anal shield, all in dorsal views (overview, anterior margin and cuticular impressions, respectively) **K–M** anal shield, underside, all in ventral views (surface, posterior margin, and overview, respectively). Scale bars in millimetres.

***Tegument*** (Figs 8A–G, 10): Dull; collum, thoracic shield, tergite and anal shield with tiny golden setae; each seta located in small pit. Anterior margins of midbody tergite and of anal shield with lower number of setae than posterior margins. Posterior rim on dorsal and ventral side of anal shield with a few small setae.

**Figure 11.**
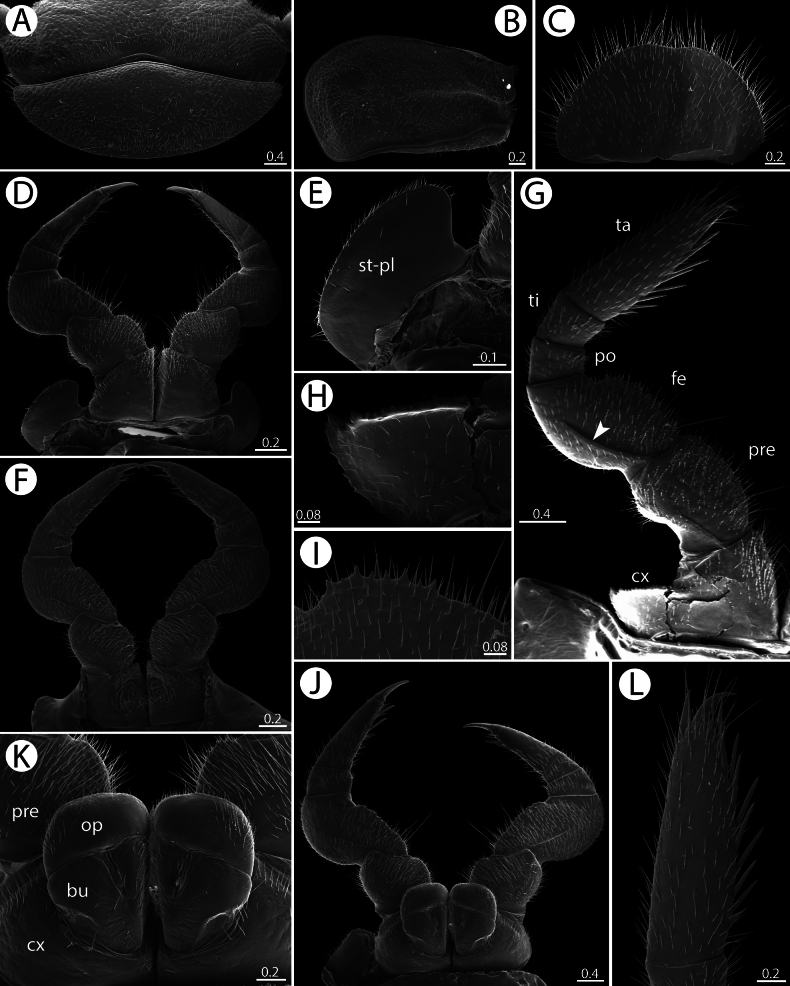
*Zephroniamacula* sp. nov. — SEM**A–G, L** ♂ paratype (CUMZ-MYR0016) **J, K** ♀ paratype (CUMZ-MYR0015) **A** collum **B** seventh pleurite **C** subanal plate **D** legs 1 **E** stigmatic plate 1 **F** legs 2 with gonopores **G** leg 10 (arrow points to femoral ridge) **H** coxal process on leg 10 **I** small teeth on femur **J** leg 2 with vulvae **K** vulvae **L** tarsus 10. Abbreviation: bu = bursa, cx = coxa, fe = femur, op = operculum, po = postfemur, pre = prefemur, st-pl = stigmatic plate, ta = tarsus, ti = tibia. Scale bars in millimetres.

***Collum*** (Figs 8C, D, 11A): Sub-semicircular; tip of lateral margin obtuse, covered with fine setae.

***Thoracic shield*** (Fig. 10B): With a shallow, large, wide groove separated by a large and long ridge. Slope towards groove without keels. Groove smooth, without setae; on a ridge with a few setae.

***Midbody tergite*** (Fig. 10D–G): With a row of oval impressions; anterior margin with tiny tubercles. Inner area with a crenate barrier; a wide and shallow groove. Tips of midbody paratergites projecting ventro-posteriad.

***Anal shield*** (Fig. 10H–M): In female large and well-rounded, in male slenderer. With a row of oval impressions, edge of anterior margin with numerous tiny tubercles (sometimes arranged into rows). Inner area with a row of oval impressions, forming a crenate barrier; with a wide and shallow groove.

***Endotergum of thoracic shield and midbody tergite*** (Fig. 14E–H): Thoracic shield similar to midbody tergites. Overall surface smooth. Posterior margin (pm) flat, not modified. Outer area (os) without setae. Bristles arranged in one row; tip of longest bristles reaching to posterior margin (Fig. 14G, H showing short bristles). Middle area (ma) with a single row of conspicuous, oval cuticular impressions; distance between impressions twice as long as individual diameter. Inner area (ia) without tubercles or setae.

***Pleurite (laterotergite)*** (Fig. 11B): Densely setose. First pleurite boomerang-shaped; apical margin attenuated; strongly projecting into sharp tip. Pleurite 2 projecting into an obtuse tip. Remaining pleurites flat and wide, lamella-like, apical margin extended.

***Subanal plate of female*** (Fig. 11C): Sparsely setose. Large and broad (sometimes quite narrow), semicircular; apical margin slightly concave (rarely truncate/obtuse).

***Stigmatic plates*** (Fig. 11D, E): First stigmatic plate large, slender; apex well-rounded, slightly curved towards basal part of prefemur. Second stigmatic plates in both male (Fig. 11F) and female (Fig. 11J) only very weakly curved towards coxa.

***Legs*** (Fig. 11D–L): All podomeres with long setae. Coxa (cx) large, as long as length of prefemur; coxal process absent in first and second legs; legs 3–21 marginally with dentate process (tooth), conspicuous, broad. Prefemur (pre) stout; apico-mesally with weak projection; mesal margin with conspicuous and small spines. Femur (fe) short and stout, as long as length of prefemur; with a long ridge in all leg-pairs; apico-mesally with strong spines, forming a dentate margin. Postfemur (po) and tibia (ti) quite short. Tarsus (ta) of midbody legs quite short; as long as length of femur+postfemur; first two leg-pairs without an apical spine; leg-pair 1 with 1 ventral spine; leg-pair 2 with 3 ventral spines; leg-pair 3 with 4–6 ventral spines and 1 apical spine; leg pairs 4 with 6–8 ventral spines and 1 apical spine; leg pairs 5–21 with 7–9 ventral spines and 1 apical spine. In leg 9, length of femur equal to width, tarsus 4× longer than wide. Claw normal, at base with a notch, conspicuous.

***Male sexual characters*** (Fig. 11F): Gonopore large, with long setae; with divided sclerotized plates, triangular.

**Figure 12.**
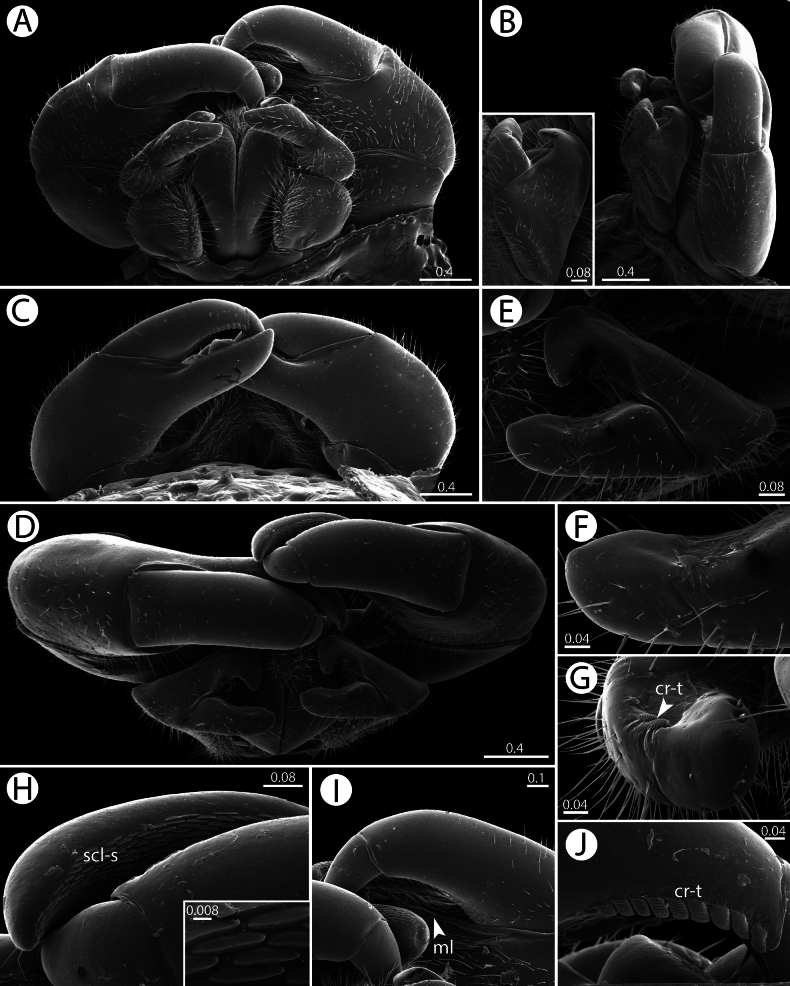
*Zephroniamacula* sp. nov., ♂ paratype (CUMZ-MYR0016) — SEM**A–D** overview of telopods (anterior view, lateral view with emphasis on anterior telopod, posterior view and ventral view, respectively) **E–G** anterior telopod (telopoditomeres 2–4, movable finger, and crenulated teeth, respectively) **H–J** posterior telopod (sclerotize spots on immovable finger, telopoditomeres 2–4 with emphasis on membranous lobe and crenulated teeth, respectively). Abbreviations: cr-t = crenulated teeth, ml = membranous lobe, scl-s = sclerotized spots. Scale bars in millimetres.

***Anterior telopods*** (Fig. 12A, B, E–G): First telopoditomere stout, rectangular. Telopoditomere 2 large. Immovable finger (process of telopoditomere 2) quite long; as long as telopoditomere 3; clearly seen in posterior and anterior views; strongly curved; tip obtuse, directed anteriad and close to basal part of telopoditomere 4; with a large membranous lobe; with sclerotized spots located at inner margin. Telopoditomere 3 longer than telopoditomere 4; clearly demarcated from telopoditomere 4 by conspicuous suture; apically with crenulated teeth (cr-t). Tepoloditomere 4 apically with a few setae located in the side pits.

**Figure 13.**
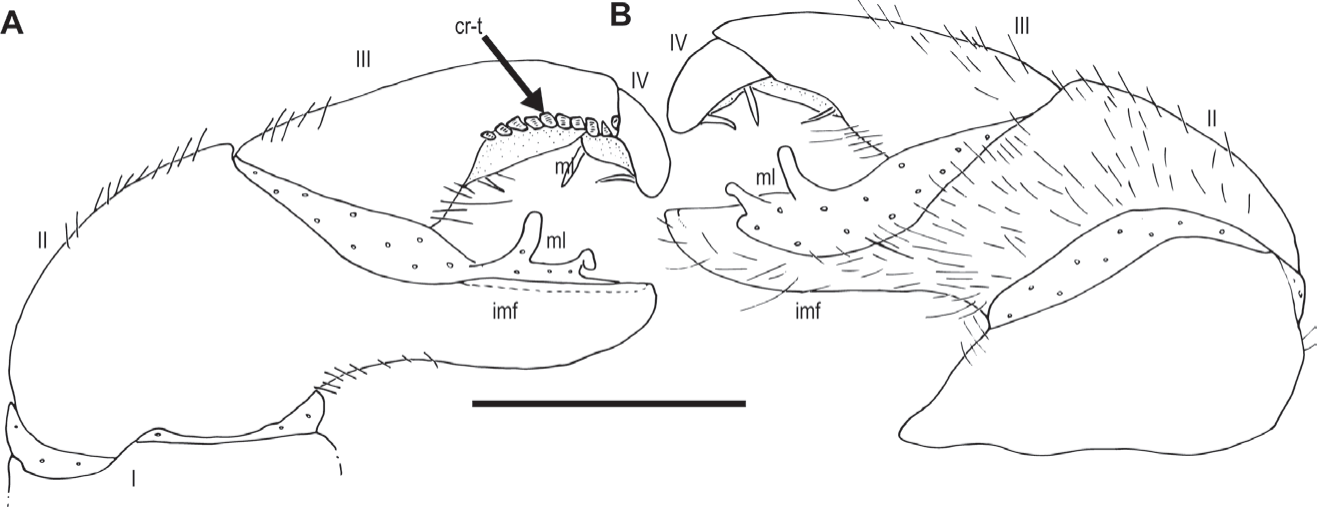
*Zephroniachantaburiensis* sp. nov., ♂ paratype (ZFMK-MYR13660), drawings — left posterior telopod **A** anterior view **B** posterior view. Abbreviations: cr-t = crenulated teeth, imf = immovable finger, ml = membranous lobe. Scale bar: 0.5 millimetres.

***Posterior telopods*** (Figs 12A–D, H–J; 13): With 4 telopoditomeres. First telopoditomere rectangular, stout; slightly longer than wide. Telopoditomere 2 large and stout. Immovable finger (process of telopoditomere 2) long and slender, 3× longer than wide; equal in length to movable finger (= combination of telopoditomeres 3+4); attenuate near tip; tip obtuse, *in-situ* curving anteriad; inner margin with conspicuous sclerotized spots (scl-s) in a blackish ledge and two large membranous lobes (ml). Telopoditomere 3 long, 2.5× longer than wide, inner margin with a long membranous ledge and basally with a single spine. Telopoditomere 4 quite short; 3.5× shorter than telopoditomere 3; slightly tapering toward apex; with two spines located on membranous ledge at inner margin; with 9 or 10 large crenulated teeth (cr-t) posteriorly. Telopoditomeres 1 mostly glabrous, telopoditomere 2 in posterior view mostly glabrous, in anterior view covered by setae. Telopoditomeres 3 and 4 in posterior view glabrous; at basal part of telopoditomere 3 in anterior view sparsely setose. Inner horns with sharp-edged tips, slightly curved caudad.

**Figure 14.**
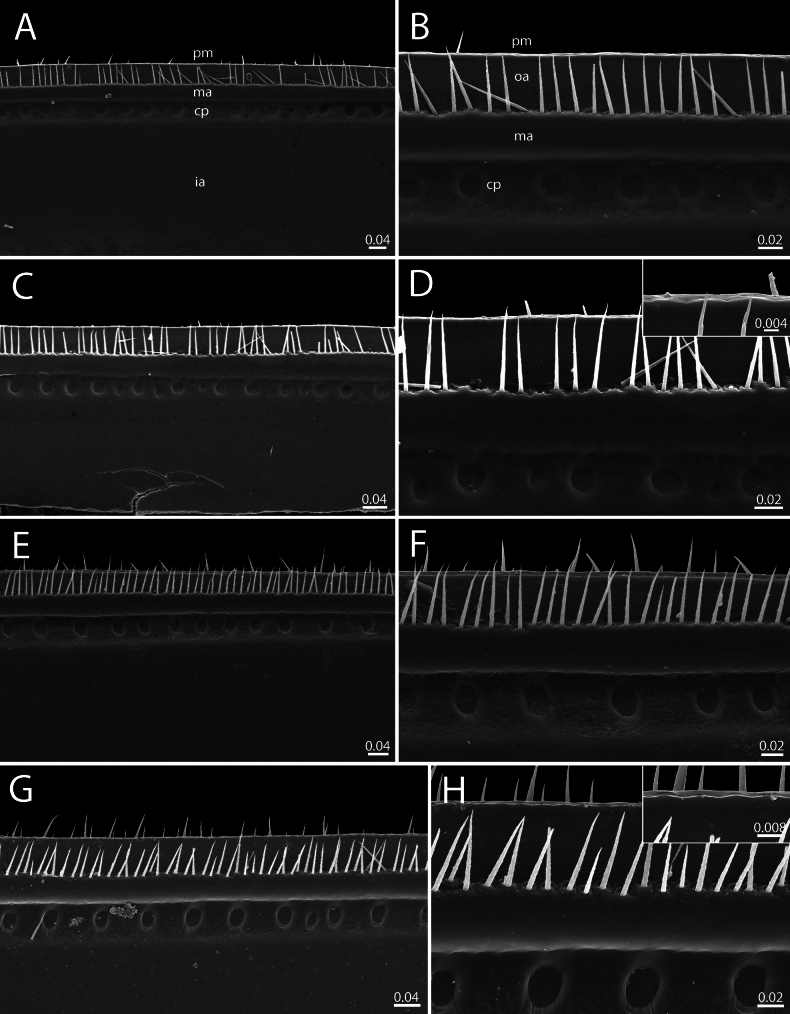
Endoterga of thoracic shields and midbody tergites — SEM**A–D***Zephroniachantaburiensis* sp. nov. (♂ paratype, CUMZ-MYR0014) **E–H***Zephroniamacula* sp. nov. (♂ paratype, CUMZ-MYR0016) **A, B, E, F** thoracic shields **C, D, G, H** midbody tergites. Abbreviations: cp = cuticular impression, ia = inner area, ma = middle area, oa = outer area, pm = posterior margin. Scale bars in millimetres.

**Figure 15.**
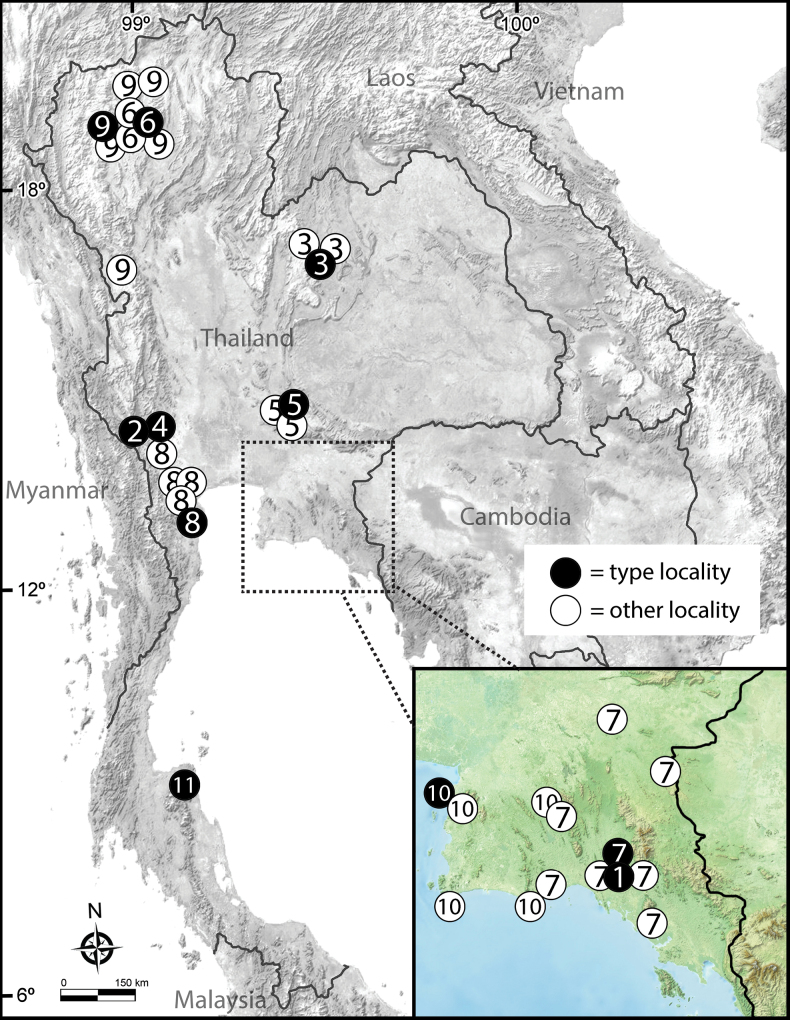
Distribution and localities of *Zephronia* species in Thailand. Number inside the circle indicates species: 1 = *Z.chantaburiensis* sp. nov.; 2 = *Z.chrysomallos*; 3 = *Z.enghoffi*; 4 = *Z.erawani*; 5 = *Z.golovatchi*; 6 = *Z.lannaensis*; 7 = *Z.macula* sp. nov.; 8 = *Z.panhai*; 9 = *Z.phrain*; 10 = *Z.siamensis*; 11 = *Z.viridisoma*.

***Female sexual characters*** (Fig. 11J, K): Vulva large and stout, sparsely setose; covering almost 1/3 of coxa; located at mesal margin; mesally extended to base of prefemur. Operculum (op) broad and well-rounded; swollen dorsoventrally, mesal margin not protruding; tip of operculum truncate. Bursa (bu) large, completely demarcated from operculum by a triangular groove.

##### Remarks.

Intrapopulational variation was found in which most specimens have a brown/reddish brown (majority) colour, while the others are greenish brown (minority). In addition, the posterior margin of subanal scale is in most specimens wide, whereas some specimens possess a quite narrow margin.

##### Distribution and habitat.

This species is widely distributed across eastern Thailand (>150 km). Most specimens were collected from locations with granitic rock habitats, while a few localities were in forested limestone habitats (Fig. 8H, I). All individuals were encountered during the day under rotten logs and in leaf litters.

##### Etymology.

The name is a noun referring to the pattern of spots on the body.

## ﻿Discussion

Two new species of giant pill-millipedes from Thailand have been integratively described on the basis of morphological characters and genetic information. These new species are taxonomically assigned to the genus *Zephronia* due to the combination of the distinct characters of the antennae, endotergum, tarsi of legs 5–21, anterior telopods, posterior telopods and female vulvae; they share a few morphological traits with most of their congeners (viz., body size ca 20 mm with brown colour, one row of bristles on endotergum). They clearly belong to the *Zephronia* s.s. species group (*Z.chrysomallos*, *Z.dawydoffi*, *Z.erawani*, *Z.enghoffi*, *Z.golovatchi*, *Z.hui*, *Z.konkakinhensis*, *Z.lannaensis*, *Z.laotica*, *Z.medongensis*, *Z.montis*, *Z.ovalis*, *Z.panhai*, *Z.siamensis* and *Z.zhouae*), both morphologically and genetically (Fig. 1). However, *Z.chantaburiensis* sp. nov. and *Z.macula* sp. nov. obviously differ from other *Zephronia* s.s. species in the presence of only a single apical spine on the tarsi, as well as in the anterior telopods (having a relatively long and strongly curved immovable finger). Among the *Zephronia* species in Thailand, the presence of a single apical spine on the tarsi is only known for *Z.viridisoma*, a species genetically distant from most *Zephronia* species (Fig. 1). One of the new species, *Z.macula* sp. nov., exhibits a distinct colour pattern by having spots/bands throughout the body tergites, which is unique for the genus, but similar to the one observed in *Sphaerobelumnigrum* Wesener, 2019 (Wesener 2019).

The interspecific distances based on the 658 bp COI barcoding fragment of the two new species compared to its congeners are quite large, with 18.94–26.82% in *Z.chantaburiensis* sp. nov. and 17.93–25.13% in *Z.macula* sp. nov. While the usual range of interspecific genetic distances for species discrimination in most giant pill-millipedes is from 8 to 21%, the observed distances in this study are higher than between most previously recognized species within the genus. Our analysis of the interspecific distances is consistent with those reported in recently described *Zephronia* species (Rosenmejer et al. 2021; Bhansali and Wesener 2022), which have interspecific distances ranging from 8–15%. The relatively high maximum interspecific distances observed between millipede species have been proposed to result from isolated distributions with different degrees of geographical barriers, because often closely related species are found geographically far from one another (Cádiz et al. 2018; Means and Marek 2017). The geographical distances between the new species described here and other *Zephronia* species also support the discrimination of our new species. Although our single-locus phylogenetic tree placed our two new species into the *Zephronia* s.s. (Fig. 1), the deep evolutionary relationships and the monophyly of this widespread and diverse genus, especially *Zephronia* s.s. species group, are still unclear. An integrative approach implementing additional genetic markers such as nuclear genes (e.g. see Dietz et al. 2023), together with the inclusion of more taxa should be pursued to clarify their generic-species status.

We conducted four intensive surveys throughout the eastern part of Thailand from 2019 to 2023. According to the distribution record, *Z.macula* sp. nov. has a wide range, covering the majority of eastern Thailand (Fig. 13). The wide distribution is similar to those of *Z.siamensis* and *Z.phrain*, which are dispersed over an area stretching 200–300 km^2^ (Srisonchai et al. 2021). The wide distribution of *Z.macula* sp. nov. is probably due to its occurrence in a wide range of granite and limestone habitats, where it is easily encountered, whereas *Z.chantaburiensis* sp. now. has been collected only in areas with granitic soils. It is important to note that *Z.chantaburiensis* sp. nov. lives in sympatry with *Z.macula* sp. nov. by sharing the same habitat/ microhabitat; individuals of both species can be found under leaf litter and rotten logs. Quite often, several millipede species of the same genus can be found in the same location, e.g., in *Zoosphaerium* Pocock, 1895; *Eviulisoma* Silvestri, 1910; *Chaleponcus* Attems, 1914 (Wesener and Sagorny 2021; Enghoff 2014, 2018). Given that the two new species were only found in the eastern part of Thailand, we here regard them as endemic.

The present work adds two endemic species of *Zephronia*, resulting in a total of 11 species for the genus in Thailand (51 valid species worldwide). This discovery has also greatly expanded the known range of the genus in the far eastern part of Thailand, but leaves a gap of more than 400 km without any record of *Zephronia* along the coast of Thailand in the closest country area (Cambodia) where the Cardamom Mountain Range lies. Searching in still unexplored places in Thailand and neighboring counties would be fruitful in revealing the hidden diversity of the genus and the evolution of the taxon. It is believed that as investigations and intensive analyses go further, more new species will certainly be discovered.

## Supplementary Material

XML Treatment for Zephronia

XML Treatment for Zephronia chantaburiensis

XML Treatment for Zephronia macula

## ﻿Additional information

### Author ORCIDs

Ruttapon Srisonchai https://orcid.org/0000-0002-7142-0999

Natdanai Likhitrakarn https://orcid.org/0000-0002-1306-317X

Chirasak Sutcharit https://orcid.org/0000-0001-7670-9540

Thomas Wesener https://orcid.org/0000-0002-2028-3541

### Data availability

All of the data that support the findings of this study are available in the main text or Supplementary Information.

## Supplementary materials

Supplementary material 1 Estimates of cytochrome c oxidase I (COI) sequence divergences (uncorrected p-distances) within and among *Zephronia* species and related genera (*Sphaerobelum*, *Prionobelum* and *Cryxus*) Data type xlsx https://binary.pensoft.net/file/1133569This dataset is made available under the Open Database License (http://opendatacommons.org/licenses/odbl/1.0/). The Open Database License (ODbL) is a license agreement intended to allow users to freely share, modify, and use this Dataset while maintaining this same freedom for others, provided that the original source and author(s) are credited.Ruttapon Srisonchai, Natdanai Likhitrakarn, Chirasak Sutcharit, Thomas Wesener

Supplementary material 2 Phylogenetic tree based on maximum likelihood (ML) approach of genus *Zephronia* and related genera (*Sphaerobelum*, *Prionobelum* and *Cryxus*) Data type pdf https://binary.pensoft.net/file/1133570This dataset is made available under the Open Database License (http://opendatacommons.org/licenses/odbl/1.0/). The Open Database License (ODbL) is a license agreement intended to allow users to freely share, modify, and use this Dataset while maintaining this same freedom for others, provided that the original source and author(s) are credited.Ruttapon Srisonchai, Natdanai Likhitrakarn, Chirasak Sutcharit, Thomas Wesener
