# Supplementary figures and images for: ﻿Integrative taxonomy reveals two new giant pill-millipedes of the genus Zephronia Gray, 1832 from eastern Thailand (Diplopoda, Sphaerotheriida, Zephroniidae)
Source: Zookeys. 2024 Sep 13;1212:29–64. doi: 10.3897/zookeys.1212.126536 (PMC11415620; doi:10.3897/zookeys.1212.126536)

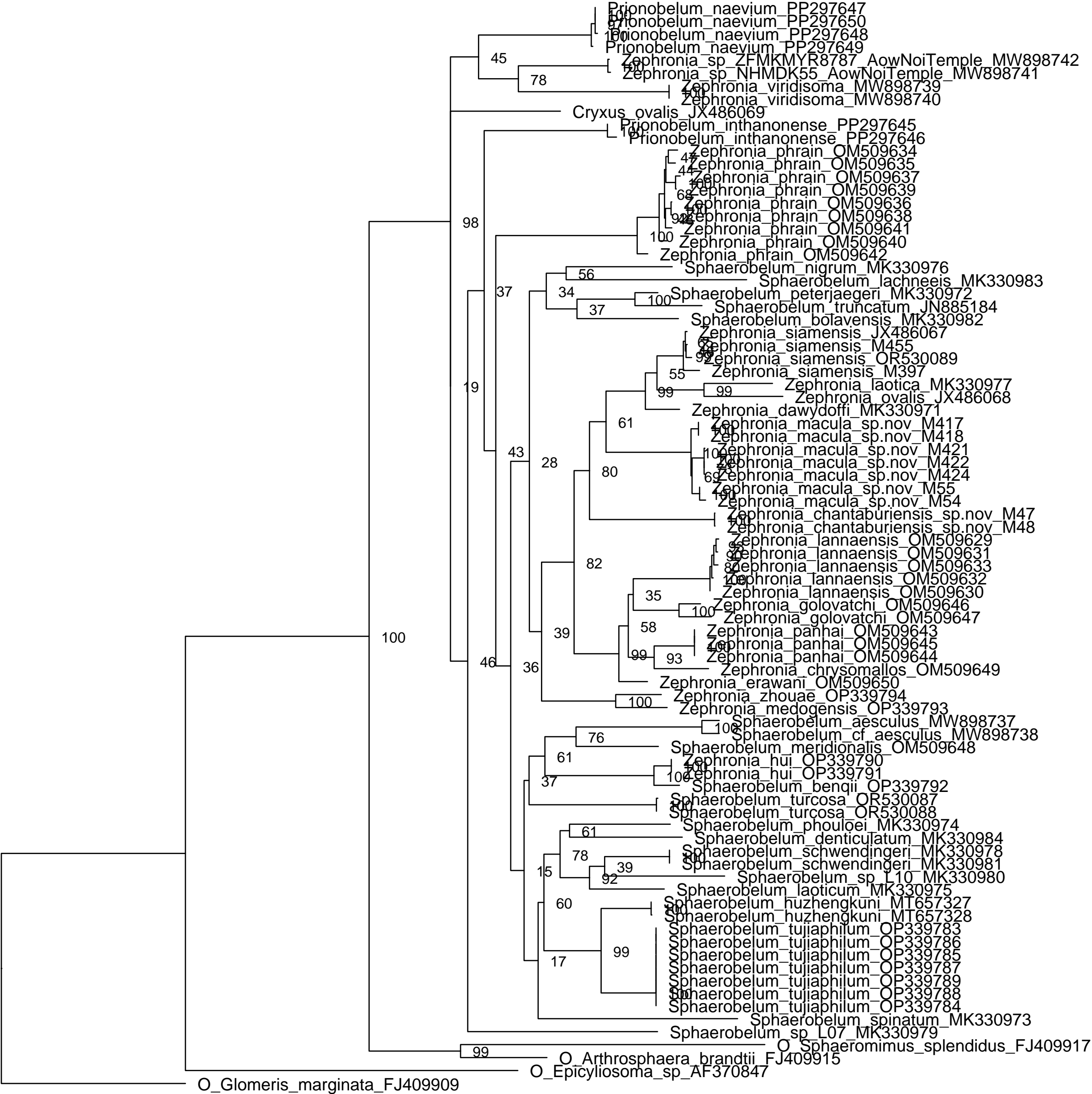

0.2

Supplement: Supplementary material 2 — Phylogenetic tree based on maximum likelihood (ML) approach of genus Zephronia and related genera (Sphaerobelum, Prionobelum and Cryxus) [file zookeys-1212-029_article-126536__-s002.pdf]
